# Mechanical properties of radish petioles and calibration of cohesion parameters in discrete element models

**DOI:** 10.3389/fpls.2025.1634962

**Published:** 2025-09-24

**Authors:** Zhendong Zhang, Guocheng Bao, Yanwei Yuan, Zhouyi Lv, Xinxin Chen, Xuedong Chen, Wei Yang

**Affiliations:** ^1^ Institute of Tillage and Plant Protection Equipment, Chinese Academy of Agricultural Mechanization Sciences Group Co., Ltd., Beijing, China; ^2^ State Key Laboratory of Agricultural Equipment Technology, Chinese Academy of Agricultural Mechanization Sciences Group Co., Beijing, China; ^3^ College of Science, China Agricultural University, Beijing, China

**Keywords:** radish petiole, harvester, biomechanical properties, discrete element, bonding parameter

## Abstract

An understanding of the biomechanical properties of radish petioles is critical for the rational design of harvesting machinery and the optimization of the harvesting process. At present, research on the biomechanical properties of radish petioles is relatively scarce, and there is a lack of bonding parameters for the discrete element simulation model of radish petioles. To address these challenges, this study explores the impact of varying petiole parts, moisture content, and tissue structure on their mechanical properties through histological analysis and torsional testing. Subsequently, a discrete element simulation model for radish petioles, suitable for mechanized harvesting processes, was developed based on the BondingV2 model. The model’s bonding parameters were optimized through Plackett-Burman and central composite experiments. The experimental results indicate that the torsional modulus of the radish petiole is significantly influenced by moisture content and tissue structure, with the highest torsional modulus observed at the petiole’s distal end, exhibiting optimal mechanical performance at intermediate moisture levels. The petiole’s distal end exhibited the following properties: unit area normal stiffness coefficient is 2×10^9^ N/m², unit area shear stiffness coefficient (3.12×10^9^ N/m²), normal strength (1.5×10¹¹ Pa), shear strength (7.5×10¹^0^ Pa), and Bonded Disk Scale (1.17). The simulation results of axial tension, torsional bending, three-point bending, and field tests exhibited errors of 4.46%, 8.8%, 0.41%, and 2.1%, respectively, when compared to the corresponding physical test results, thereby validating the reliability of the bonding parameters calibrated for the distal petiole of radish at the optimal moisture content. The findings of this study provide a theoretical foundation and technical support for the optimization of mechanized harvesting equipment for radishes.

## 1 Introduction

Radish (Raphanus sativus L.) is an important cruciferous crop worldwide, rich in a variety of vitamins. The global cultivated area of radish is approximately 1,096 thousand hectares, with its primary distribution in East Asia, including countries such as China, South Korea, and Japan (China Report Hall, 2025; Bao et al., 2019). Currently, radish harvesting primarily relies on manual labor, with a low level of mechanization, which significantly hinders the development of the radish industry. As the root is the main edible part of the radish, past research has predominantly focused on the root, while studies on the petiole have been relatively ne-glected. However, the influence of the radish petiole on the efficiency of mechanized harvesting is more pronounced. During the gripping and extraction of the radish petiole, the mechanical properties of the petiole do not align well with the machinery’s interaction, resulting in a higher incidence of missed harvests. Therefore, studying the biomechanical properties of the radish petiole is of significant importance for the design of high-quality radish harvesting machinery (Xiao et al., 2023).

In order to guide the design of harvesting machinery, reduce operational loss rates, and enhance operational quality, researchers have conducted in-depth studies on the biomechanical properties of various crops, including rice stems, wheat stems, sugarcane, safflower, maize stubble, and tea leaves, which have played a crucial role in agricultural production activities (Tang et al., 2022;Leblicq et al., 2015; Xie et al., 2020; Zhang et al., 2024; Zhang et al., 2023; Fu et al., 2024). However, the mechanized harvesting method for radishes differs from those of the aforementioned crops. Traditional biomechanical experiments primarily focus on the bending strength of plant stems, whereas the mechanized harvesting of radish petioles places greater emphasis on torsional strength and maximum pulling force. Furthermore, these studies have not explored the stem structure at the microscopic level nor summarized the factors influencing the mechanical properties of plant stems.

With the rapid advancement of computer simulation technology, discrete element method (DEM) simulation techniques have been widely applied in the modeling of plant stems and petioles (Tian et al., 2023). Using the discrete element method (DEM), a stretching model for tobacco leaves was developed, with calibration and verification of model accuracy, providing a foundation for the design and optimization of tobacco harvesters. This method is also applicable to studies on other plant leaves (Zhao et al., 2023). By combining the discrete element method (DEM) with mechanical experiments, a biomechanical model for cotton stalks was established, and model parameters were calibrated. The mechanical behavior of cotton stalks during compression, bending, and shear processes was simulated, providing theoretical support for the design of cotton stalk harvesting and processing machinery (Liu et al., 2022). Utilized the discrete element method (DEM) to establish a flexible model for taro plants, validating the model’s accuracy through clamping and traction experiments, thereby providing a theoretical foundation for the design and optimization of taro harvesting machinery. (Cao et al., 2024) Established and calibrated a discrete element model for the stem of the sea buckthorn plant. Through three-point bending, radial compression, and shear tests, the optimal parameter combination was determined. The simulation model effectively reflected the bio-mechanical properties of sea buckthorn, providing valuable insights for crop-machine interaction studies and the design of harvesting equipment (Hu et al., 2023). Investigated the contact parameters of hemp stalks using the discrete element method (DEM), measuring the physical parameters of hemp stalks through experiments and calibrating the contact parameters using stack angle tests. A discrete element model for hemp stalks was eventually established, providing technical support for simulating the hemp peeling process (Liu, 2018). The aforementioned studies demonstrate the feasibility of using the discrete element method to establish plant stem models. Yan et al. (2023) developed a radish model and analyzed the effects of soil compaction, pulling speed, and pulling angle on the radish extraction force. By comparing simulation experiments with actual physical tests, more accurate plant stem models can be established to study failure mechanisms and guide harvester design. Compared with repeated physical experiments, using simulation models allows for faster validation of design outcomes and reduces development costs. Existing studies on radish simulation models have primarily focused on the interaction between the radish root and soil resistance. However, there is still a lack of in-depth research on the biomechanical properties of radish petioles. To date, studies specifically targeting the modeling of radish petioles remain largely unexplored. This study focuses on the radish petiole and addresses the modeling requirements prior to the harvesting operation. It investigates the mechanical properties of petioles from different sections under varying moisture contents. Through torsion tests and histological analysis, the mechanical characteristics of radish petioles are revealed, and a mechanical model suitable for mechanized harvesting is established. In addition, discrete element simulation technology is employed to construct a simulation model of the interaction between radish petioles and harvesting equipment, providing theoretical support for the optimization of the clamping mechanism. This research aims to improve key aspects of mechanized radish harvesting by reducing the rates of missed harvests and mechanical damage, enhancing harvesting efficiency and quality, and offering a scientific basis for the future design of agricultural machinery.

## 2 Materials and methods

### 2.1 Structure of the gripping and pulling radish harvester

**Figure 1:**
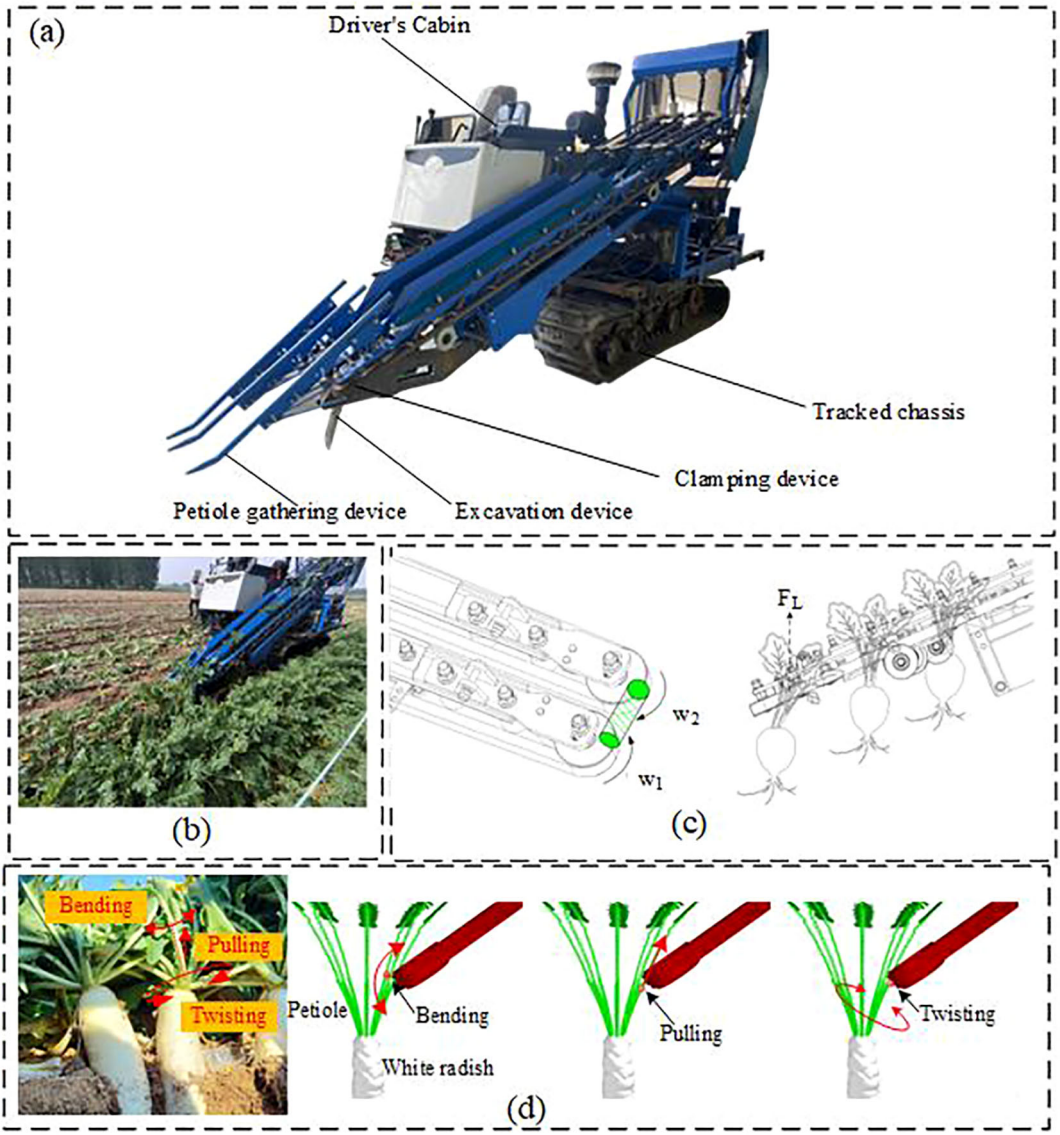
Schematic diagram of the double-row radish harvester operation. **(a)** Schematic diagram of the radish harvester structure. **(b)** Field harvesting situation of the harvester. **(c)** Schematic of the Actual Radish Harvesting Process. **(d)** Force Distribution on the Petiole During Harvesting.

As shown in **Figure 1**, the clamping-and-pulling radish harvester is capable of harvesting double-row radishes in a single operation. The front end of the pulling and clamping device is equipped with a petiole-gathering mechanism, and belts are mounted on the clamping and supporting pulleys. The clamping force is generated by the friction between two counter-rotating belts and the petiole. During the clamping process, the clamping and pulling device applies torsional torque and axial tensile force to the radish petiole, which are the primary causes of petiole rupture, resulting in a higher incidence of missed harvests.

Relevant literature and practical studies indicate that the moisture content and clamping position of the petiole are key factors affecting its physical and mechanical properties. However, research specifically focusing on radish petioles is limited. Therefore, this study aims to explore the impact of moisture content and gripping position on the mechanical properties of radish petioles. Based on radish petiole parameters suitable for mechanized harvesting, a discrete element petiole model is established, and petiole bonding parameters are calibrated to observe the interaction of the soil-plant-machine system, providing theoretical support for the design of the clamping and pulling mechanism.

### 2.2 Material preparation

**Figure 2:**
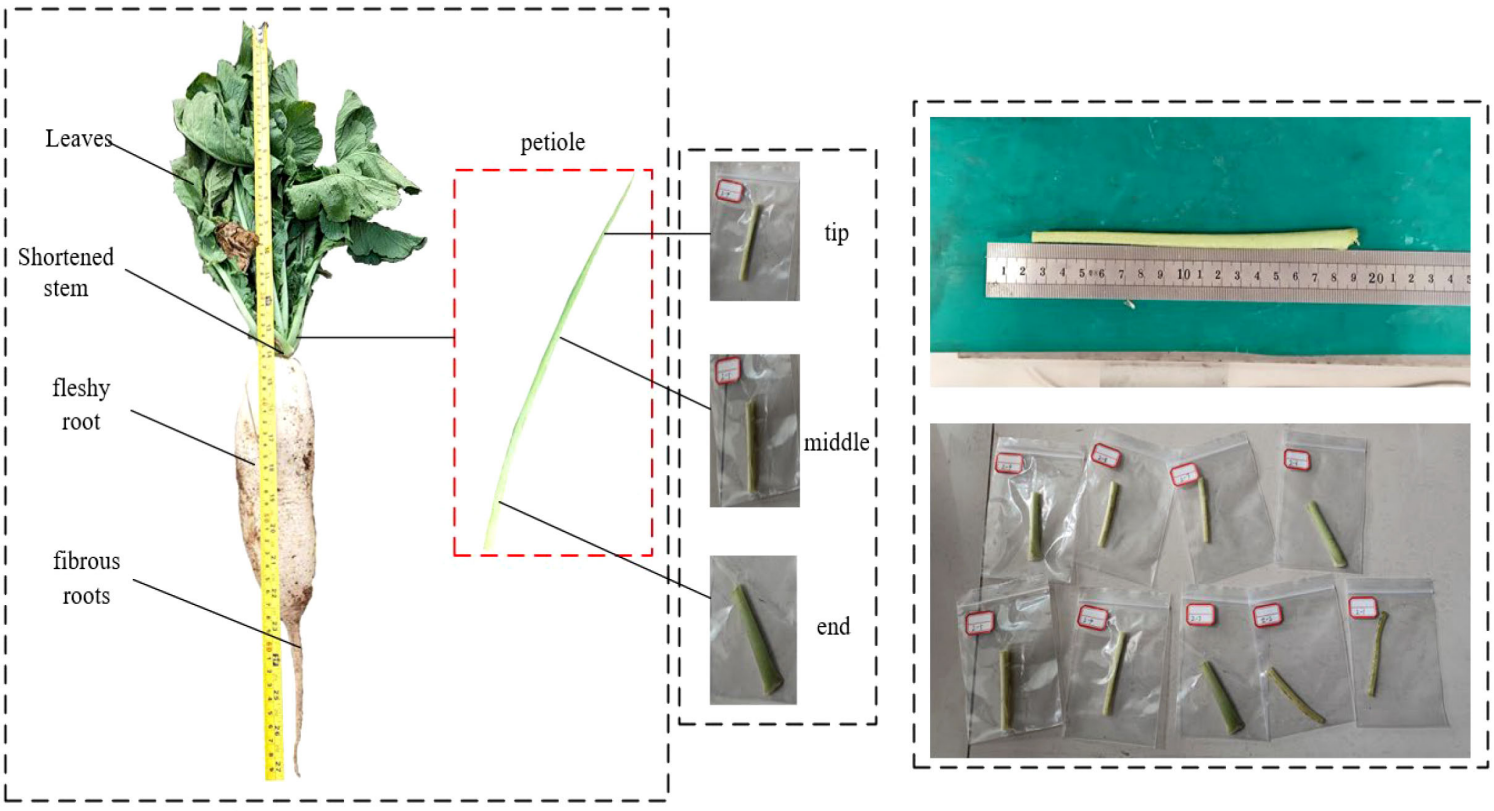
Radish composition and sampling.

Petioles of the locally cultivated radish variety Arctic Snow were collected using a five-point sampling method in Langfang, Hebei Province, China. To minimize biological variability among samples, petioles of similar growth stage and size were selected exclusively from healthy plants exhibiting vigorous growth and free from pests and diseases. The leaf blades were carefully pruned to remove excess foliage, retaining only the petiole stems necessary for specimen preparation. Segments of 70 mm in length were excised from the end, middle, and tip of each petiole and used for tensile, torsional, and three-point bending tests, as illustrated in **Figure 2**. To ensure the accuracy of the mechanical data, all tests were conducted within 48 hours of sample collection.

### 2.3 Histological analysis of radish petioles

**Figure 3:**
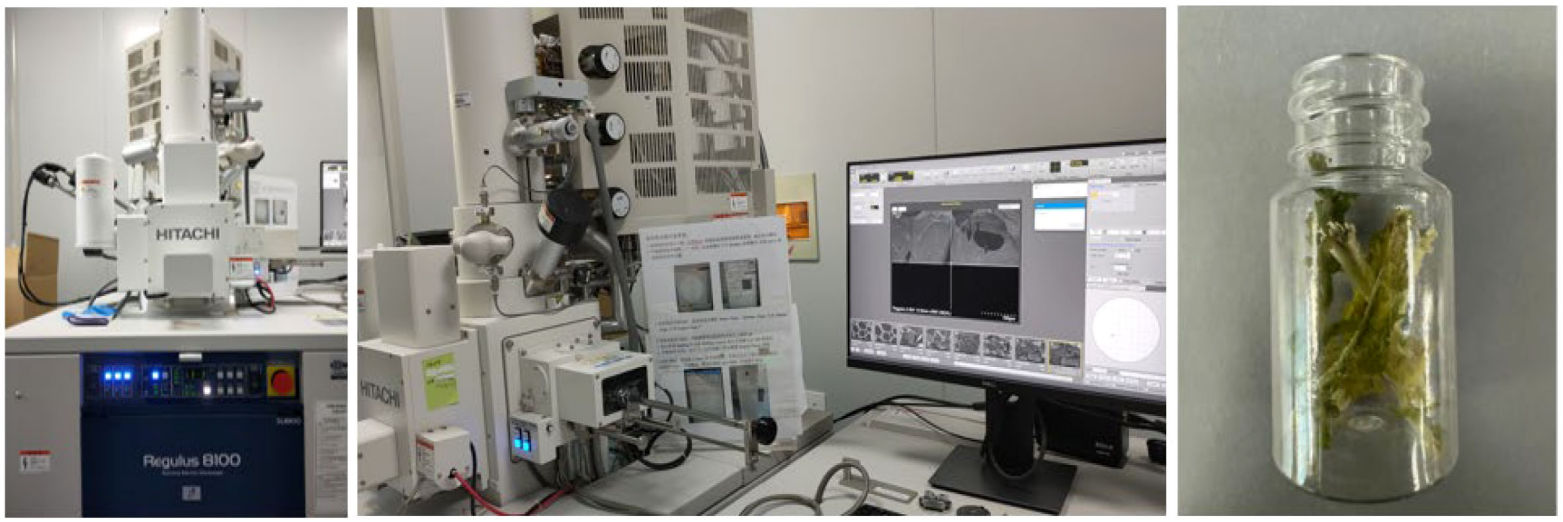
Testing apparatus and experimental accessories for petioles.

The petiole can be regarded as a hierarchical structure with structural features defined at multiple scales. The petiole is typically composed of various cell tissues, and its mechanical properties mainly depend on the geometric shape, structural characteristics of the compositional components, and the microscopic structure of the tissues (Shah et al., 2017). To analyze the impact of the internal structure of the petiole on its mechanical properties, an electron microscope was used to observe the cross-sectional morphology of the petiole. The distribution of various cellular tissues, including the epidermis, collenchyma tissue, parenchyma tissue, and vascular tissue, was studied. (Langer et al., 2021; Faisal et al., 2013) The radish petiole was processed using a dehydration followed by liquid nitrogen quenching method to prepare the samples, which were then observed using the Regulus 8100 electron microscope (Hitachi High-Tech), as shown in **Figure 3**.

**Figure 4:**
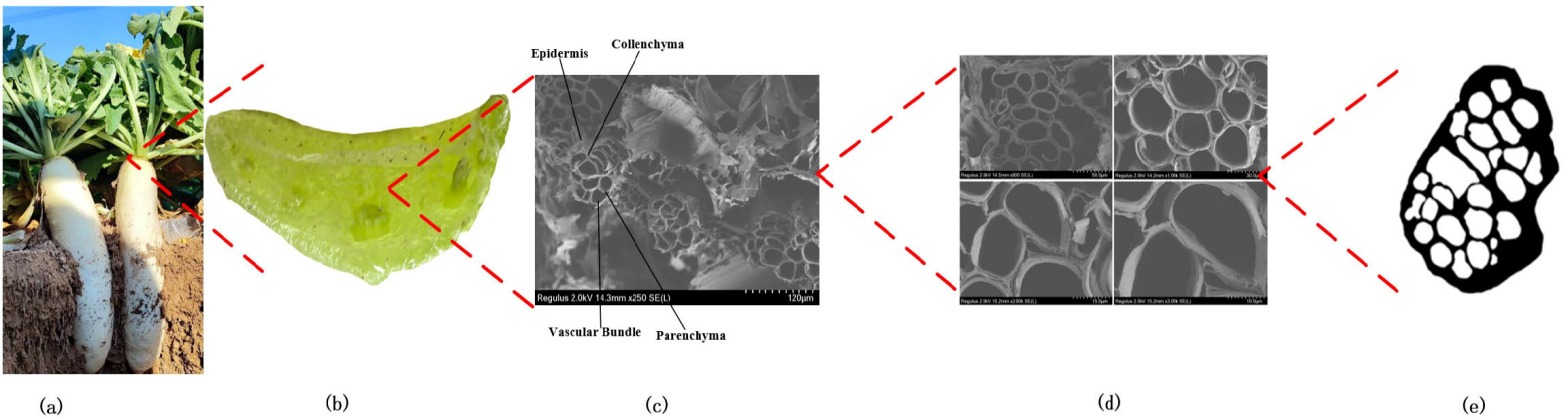
Hierarchical structure of the radish petiole. **(a)** Radish. **(b)** Petiole cross-section with multiple tissue. **(c)** Tissue microstructure. **(d)** The microstructural morphology of petiole cross sections at different magnifications. **(e)** The cross-section after morphological processing.

As shown in **Figure 4**, the petiole is a porous, honeycomb-like composite structure composed of vascular bundles and the surrounding parenchyma and collenchyma tissues. The outermost layer is the epidermis, which occupies the smallest volume of the petiole. Beneath the epidermis, there is a densely arranged collenchyma tissue that is tough, plastic, and extensible. It is tightly connected to the epidermis, providing primary support and protection to the petiole, with strong resistance to compression, tension, and torsion. The region between the collenchyma tissue and the vascular bundles is primarily composed of parenchyma cells. The parenchyma cells spread from the edges towards the xylem, enveloping the vessels and dividing them into different regions, forming the vascular bundles. As shown in **Figures 4c, d**, the vascular bundles are arranged in a spiral pattern, resembling a spring, and are connected to the parenchyma tissue. This arrangement facilitates the transport of nutrients and simultaneously enhances the petiole’s ability to undergo elastic deformation during torsion.

The vascular bundle density of the petiole is a crucial factor influencing its mechanical properties. As illustrated in **Figure 4e**, the microscopic structure of the petiole was morphologically processed, and its cross-section was analyzed using OpenCV. The vascular bundle area ratio was calculated, and the vascular bundle area proportions at the tip, middle, and end were statistically determined, as shown in **Table 1**.

**Table 1:** Proportional area of vascular bundles in different parts.

Parts	Proportion of Area Occupied by Vascular Bundles
Tip	73.17%
Middle	65.52%
End	51.4%

### 2.4 Mechanical property calculation of radish petioles.

**Figure 5:**
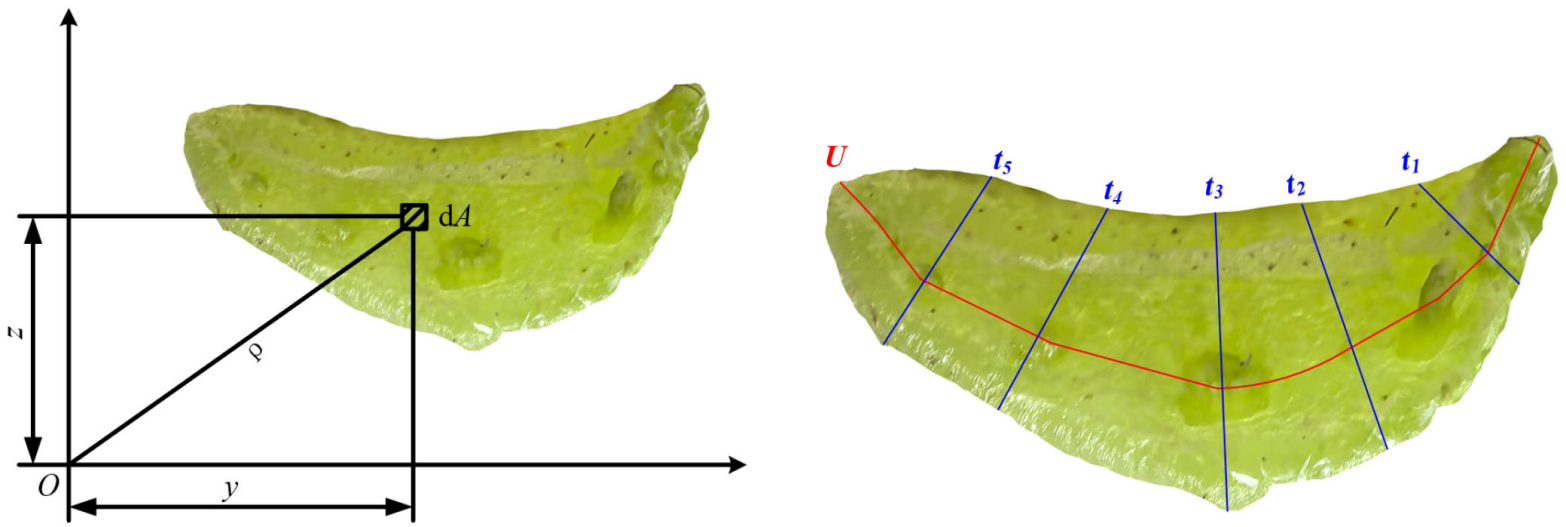
Calculation of the polar moment of inertia for the petiole cross-section.


The polar moment of inertia has a significant impact on mechanical behaviors, such as the torsional modulus. As shown in **Figure 5**, the moment of inertia of the petiole cross-section with respect to the Y-axis and Z-axis is determined through SEM images and plotting software. The formula for calculating the moment of inertia is shown in Equation 1:

(1) $$\left\{ \begin{array}{c} I_{y}={\text{∫}}_{A}z^{2}dA\\ I_{Z}={\text{∫}}_{A}y^{2}dA \end{array}\right.$$

Utilizing the moments of inertia of the petiole cross-section relative to the Y-axis and Z-axis, denoted as $I_{y}$ and $I_{z}$ respectively, the polar moment of inertia can be derived, as shown in Equation 2. This computation is grounded in the application of the Pythagorean theorem:


(2) $$\rho^{2}=y^{2}+z^{2}$$

By incorporating the values into the polar moment of inertia computation formula (Equation 3), the following is derived:


(3) $$J={\text{∫}}_{A}\rho^{2}dA={\text{∫}}_{A}z^{2}dA+{\text{∫}}_{A}y^{2}dA=I_{y}+I_{z}$$

It is established that the polar moment of inertia of the radish petiole cross-section, relative to the coordinate origin, equates to the summation of its moments of inertia about the Y-axis and Z-axis, denoted as $I_{y}$ and $I_{z}$ , respectively. Given that the cross-section of the radish petiole approximates a U-shaped configuration, the radius parameter employed in the torsion experiment is defined as the equivalent radius of the petiole, r_eq_. The computation of this equivalent radius is governed by Equation 4:


(4) $$r_{\text{eq}}=\sqrt{\frac{J}{A}}$$

The torsion modulus, a physical parameter delineating a material’s capacity to withstand deformation under torsional stress, is conventionally represented by the symbol G_k_. Typically, this modulus may be determined through the application of Equation 5 as follows:


(5) $$G_{k}=\frac{Lb}{K}\times 10^{6}$$

In the equation, *L* is the petiole length in millimeters (mm), *K* is the torsion coefficient (mm^4^), b is the slope of the torque angle(N·mm). The calculation formula for the torsional constant K of the U-section of the radish petiole is given in Equation 6.


(6) $$K=\frac{1}{3}\cdot U\cdot t^{3}$$

In the equation, U is the centreline length of the U-section (mm), t is the average thickness of the U-section collection (mm).

### 2.5 A study on the torsional mechanical properties of petiole components under different moisture content conditions

To investigate the mechanical properties of petiole components under different moisture content conditions, a series of torsional mechanical performance experiments were designed for petioles with varying moisture levels. This study established three moisture treatment groups: the low moisture group (I), the moderate moisture group (II), and the High moisture group (III). The moisture content of each treatment group was adjusted by controlling irrigation practices during the harvest period, while other growth conditions (such as light, temperature, and soil type) were kept constant.

**Figure 6:**
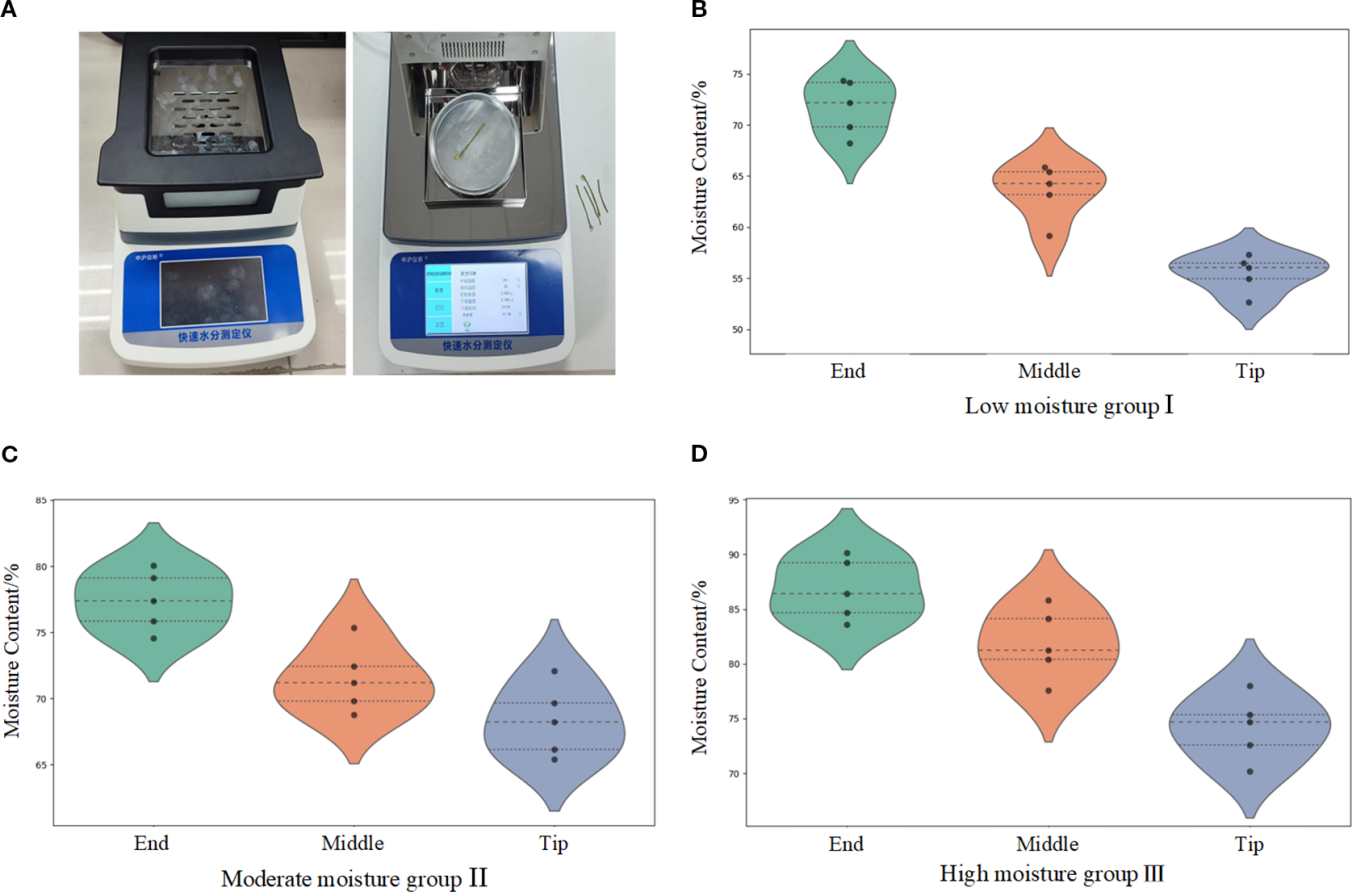
Moisture content determination apparatus and distribution of moisture content in the petiole. **(A)** Measurement process of water content in petioles. **(B)** Water content range of petioles from different parts in the low moisture group. **(C)** Water content range of petioles from different parts in the moderate moisture group. **(D)** Water content range of petioles from different parts in the high moisture group.

Samples from the distal, middle, and apical regions of the petioles were selected from groups I, II, and III, respectively. As shown in **Figure 6A**, after weighing the samples using a rapid moisture meter (Zhonghu Yixin Rapid Moisture Analyzer), they were subjected to a 20-minute drying process. The measurement results are presented in **Figures 6B–D**.

As shown in the figures, the average moisture content of the petiole’s distal region ranges from 71.72% to 86.81%, the middle region ranges from 63.57% to 81.83%, and the apical region ranges from 55.5% to 74.16%. The moisture content of the petiole components follows the trend: distal region > middle region > apical region, showing a gradual decrease from the distal to the apical regions. Analysis indicates that water in the radish plant is primarily absorbed by the roots and transported upward through the petiole. Since the distal region of the petiole is closest to the roots, it receives a more abundant water supply. Furthermore, the petiole undergoes transpiration through stomata, where water evaporates from the leaf surface, leading to a gradual reduction in moisture content at the apical region.

**Figure 7:**
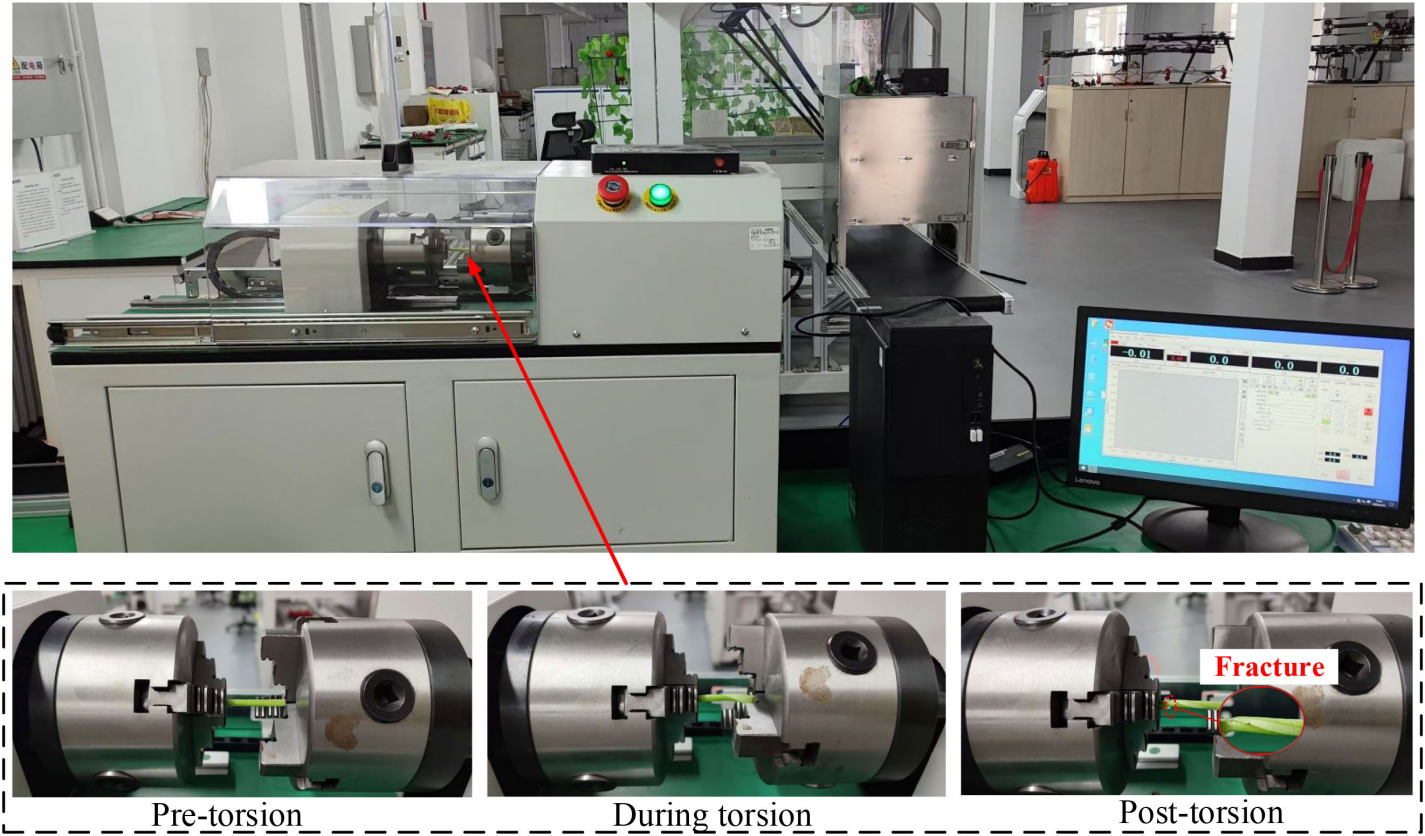
Petiole torsion test.

Torsional bending experiments were conducted to analyze the torsional fracture behavior of petioles at different regions and moisture contents, with the aim of determining the optimal clamping and extraction position, thereby laying the foundation for the subsequent development of a discrete element model for the petiole. Considering the practical operating conditions where the clamping pulleys rotate in opposite directions, generating both left-handed and right-handed torques on the petiole, the torque experiments were conducted for both left-handed and right-handed rotation scenarios. During the clamping and extraction process of the radish petiole, torsional stress is induced by the gravitational force of the leaf and external loads. Typically, the distal end of the petiole is fixed to a shortened stem, which is considered as the fixed end. Therefore, in the torsion experiment, one end is fixed while torque is applied at the other end. The torsion testing machine model is LYWN-W5N (Jinan Lingyue Precision Instruments Co., Ltd.), with the control end set to a torsional angle of 360°/min. The torque-rotation angle curve is observed, and the torsional modulus of the petiole is analyzed. The sample tests and equipment are shown in **Figure 7**.

**Figure 8:**
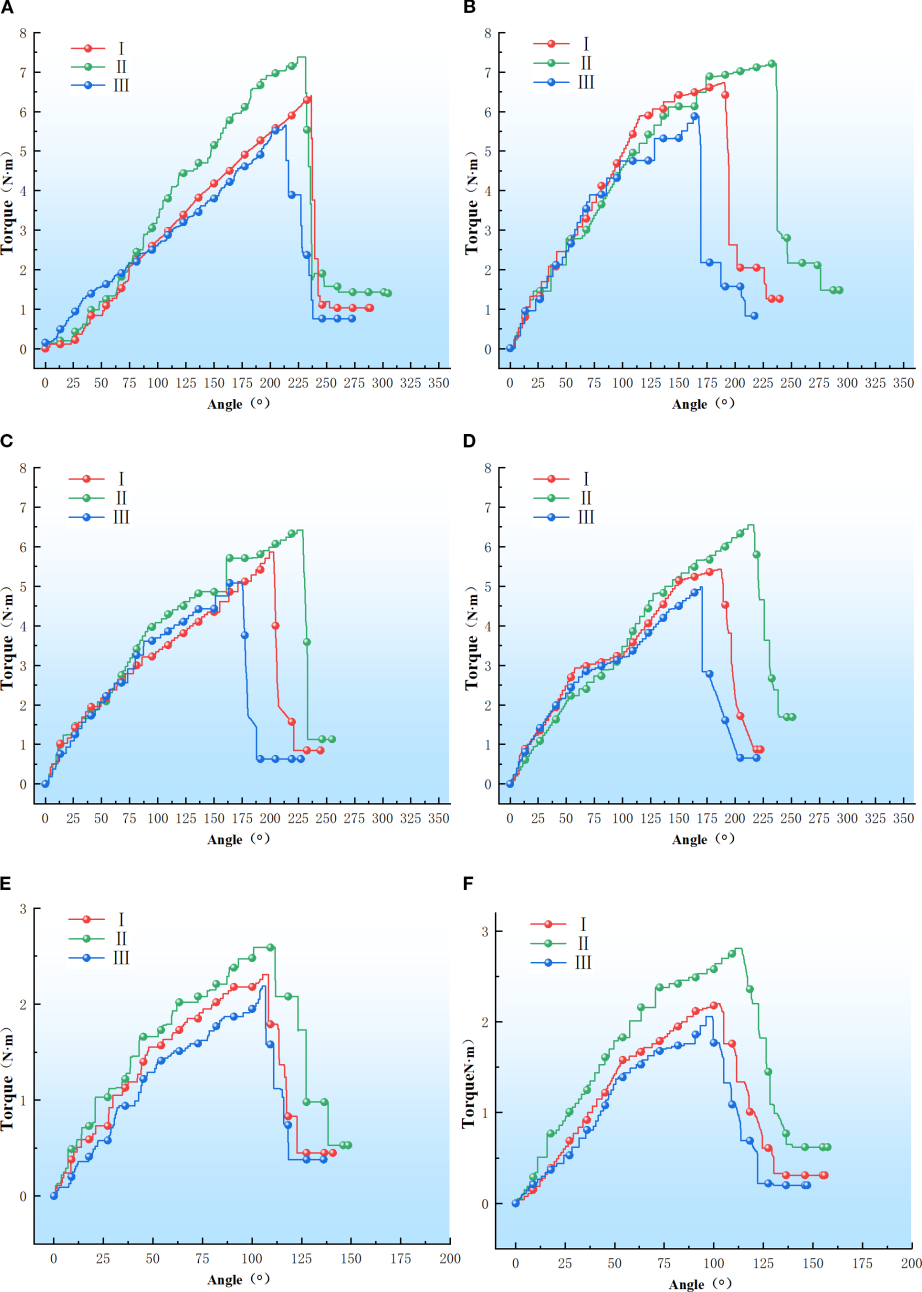
Petiole torque-angle test results. **(A–F)** Refer to the petiole end with left rotation, the petiole end with right rotation, the petiole middle with left rotation, the petiole middle with right rotation, the petiole tip with left rotation, and the petiole tip with right rotation.

Upon completion of the experiment, data from the same batch were preserved and the torque-angle curve was exported via the host software, as illustrated in **Figure 8**. The torsional modulus of the petiole is presented in **Table 2**.

**Table 2:** Discrete element model contact parameters.

Parameter		Result
Petiole-Petiole	Coeffcient of restitution	0.3
	Coeffcient of static friction	0.53
	Coeffcient of rolling friction	0.4
Petiole-Rubber	Coeffcient of restitution	0.2
	Coeffcient of static friction	0.52
	Coeffcient of rolling friction	0.3

### 2.6 Analysis of the torsion test results

As shown in **Figure 8**, the torque-angle curve of the petiole exhibits an approximately linear relationship during the initial phase, stabilizing after reaching the torsion limit. A sharp decline in torque occurs as the epidermis and vascular bundles fracture. The maximum torque values for the petiole tip in the left-handed rotation groups I, II, and III were 7.38 N·m, 6.39 N·m, and 5.65 N·m, respectively; for the right-handed rotation groups I, II, and III, the maximum torque values were 7.21 N·m, 6.73 N·m, and 5.88 N·m, respectively. For the petiole midsection, the maximum torque values for the left-handed rotation groups I, II, and III were 6.42 N·m, 5.86 N·m, and 5.08 N·m, respectively; for the right-handed rotation groups I, II, and III, the maximum torque values were 6.55 N·m, 5.43 N·m, and 4.98 N·m, respectively. For the petiole end, the maximum torque values for the left-handed rotation groups I, II, and III were 2.59 N·m, 2.31 N·m, and 2.19 N·m, respectively; for the right-handed rotation groups I, II, and III, the maximum torque values were 2.81 N·m, 2.20 N·m, and 2.06 N·m, respectively.

**Figure 9:**
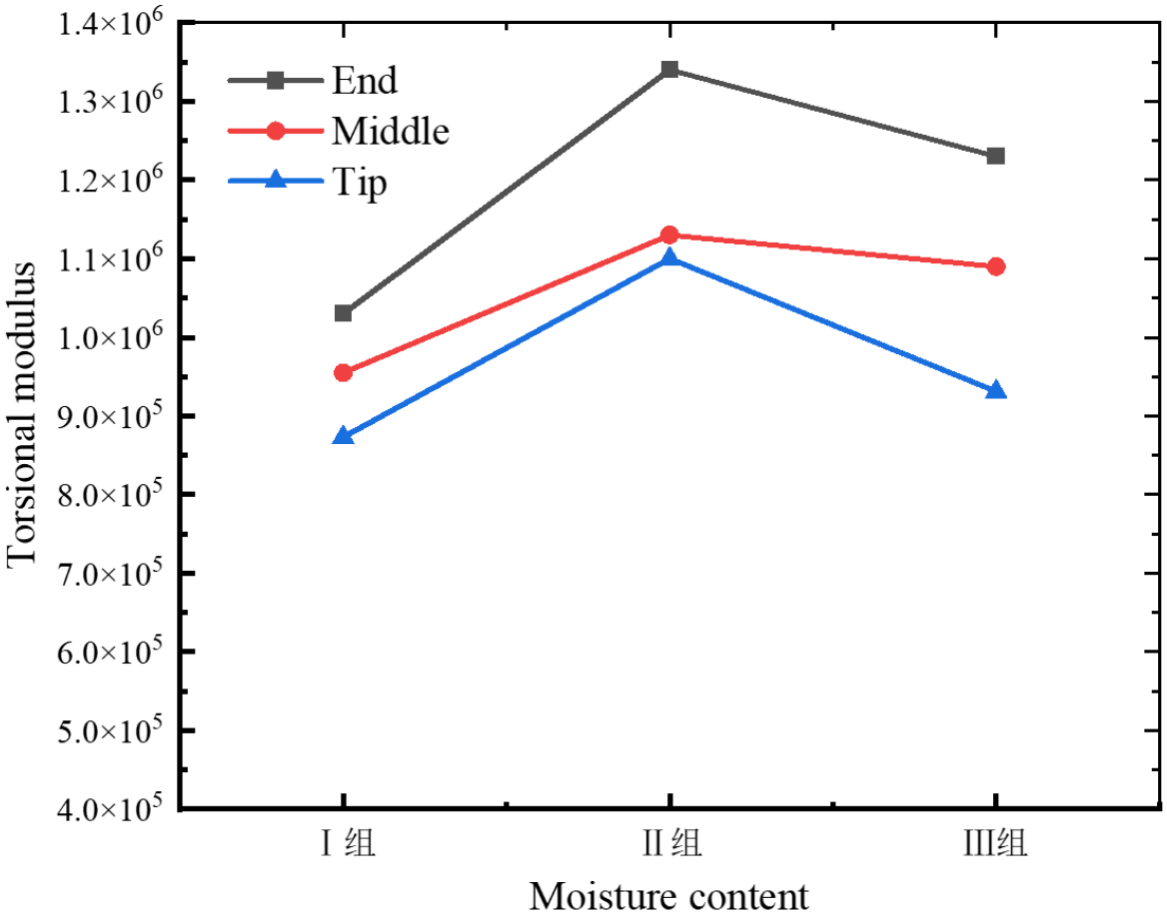
Torsional modulus of petioles at different regions under varying moisture content.

As illustrated in **Figures 8**, **9**, the torsional modulus of the petiole is lowest in Group I (low moisture content), increases in Group II (medium moisture content), and then decreases again in Group III (high moisture content). This trend indicates that the petiole exhibits lower resistance to torsional deformation under both low and high moisture conditions, while demonstrating the highest torsional stiffness at medium moisture content. In terms of petiole position, the torsional modulus is highest at the base, followed by the middle section, and lowest at the tip. This distribution is influenced by the volume fraction of vascular bundles, which affects the torsional modulus, and by the petiole’s geometric structure, which impacts the maximum torque. The base of the petiole is thicker, has the largest equivalent diameter, and contains the highest proportion of vascular bundles. As a result, both the torsional modulus and the maximum torque decrease progressively from the base to the tip. Therefore, in practical harvesting operations, the base of the petiole is the most suitable location for gripping and extraction.

As shown in **Figure 8**, the maximum fracture torque of the petiole exhibits a trend of first increasing and then decreasing with increasing moisture content, with Group II showing the highest torsional strength, followed by Group I and Group III. The analysis indicates that an increase in moisture content leads to the expansion of plant cells, thereby reducing the elasticity modulus of the cell wall, which makes the petiole more susceptible to fracture. Furthermore, excessively high moisture content reduces the stiffness of the petiole, making it more prone to failure under external forces. Moderate moisture content enhances the petiole’s toughness, preventing brittle fracture when subjected to impact or bending. Water plays a lubricating role in the cell wall structure, facilitating smoother sliding between components such as cellulose, reducing friction and stress concentration. Conversely, when the moisture content is too low, the loss of cellular water causes a decrease in turgor pressure, leading to separation of the cell membrane from the cell wall and a relaxation of the cells. Low moisture content can result in localized stress concentrations in the petiole, which, if they exceed a critical threshold, may lead to brittle fracture. Therefore, during actual harvesting, the moisture content of the petiole should be kept at an optimal level, neither too high nor too low.

## 3 Discrete element model

### 3.1 Contact model and bonding model

Based on the aforementioned research, it was determined that the distal end of the radish petiole, with a moisture content ranging from 63% to 80%, is more suitable for grasping and detachment. Additionally, the biomechanical properties of the petiole were further investigated. To enhance the theoretical framework of mechanized radish harvesting and to elucidate the interaction mechanisms between the petiole and the harvesting machinery, simulations were conducted using the EDEM software. The radish petiole model was established utilizing the BondingV2 model and the Hertz-Mindlin contact model. Employing the three-point bending test force (F_b_) as the evaluation index, significant factors were identified through a Plackett-Burman experiment. Subsequently, a mathematical model relating Fb to these factors was constructed using a central composite design, leading to the identification of the optimal parameter combination. Finally, based on the optimal parameter set, the petiole bonding parameters were configured, and the reliability of the discrete element model was validated by comparing the simulation results with experimental data.

**Figure 10:**
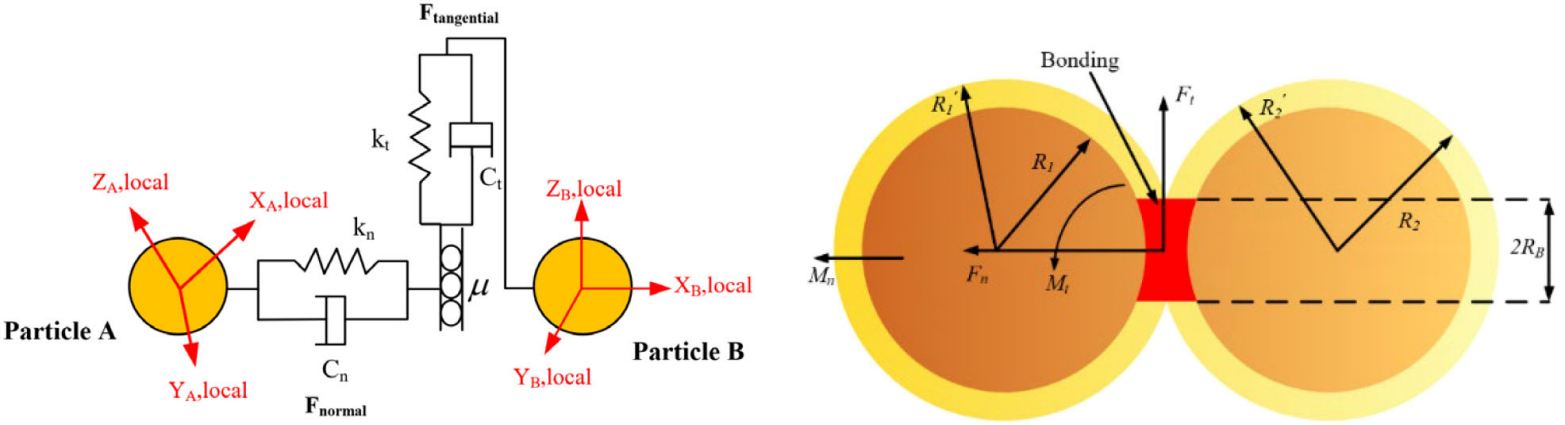
Schematic diagram of discrete element system Hertz-Mindlin model and bondingV2 model.


The Hertz-Mindlin model is employed to characterize the interactions between discrete element particles, whereas the BondingV2 contact model facilitates the cohesion of particles. The connecting bonds are capable of resisting both tangential and normal forces until the maximum normal and tangential forces are reached, at which point the bonds fracture. The Hertz-Mindlin model and the BondingV2 contact model are illustrated in **Figure 10**. During the harvesting process, the petioles of the radishes simultaneously experience both normal and tangential forces. In the BondingV2 model, the inter-particle interactions are represented through connecting bonds, with the force acting on a particle as described by Equation 7.

(7) $$\left\{ \begin{array}{c} \delta F_{n}=-\nu_{n}S_{n}A\delta t\\ \delta F_{t}=-\nu_{t}S_{t}A\delta t\\ \delta M_{n}=-\omega_{n}S_{t}J\delta t\\ \delta M_{t}=-\omega_{t}S_{n}\frac{J}{2}\delta t\\ A=\pi {R_{B}}^{2}\\ J=\frac{1}{2}\pi {R_{B}}^{4} \end{array}\right.$$

Where, *A* is the contact area (m^2^), *S_t_* and *S_n_* are the tangential and normal stiffness (N/m²), *v_t_* and *v_n_* are the tangential and normal velocities(m/s), $\omega_{t}$ and $\omega_{n}$ are the tangential and normal angular velocities(rad/s), δt is the simulation time step (s), $R_{B}$ is the contact radius of the BondingV2 (mm), $\delta F_{n}$ and $\delta F_{t}$ represent the forces in the normal and tangential directions generated by the bonding link(N), $\delta M_{n}$ and $\delta M_{t}$ represent the torques in the normal and tangential directions generated by the bonding (N·m).

The particles are bonded together through the BondingV2 adhesive bonds, where normal and shear stresses are transferred through the bonding connections. When the applied stress exceeds predefined thresholds, the BondingV2 bond fractures, as illustrated in **Figure 10**. Throughout each simulation timestep, the BondingV2 bonds persistently exert cohesive forces, which accumulate progressively over the course of the simulation. These cohesive forces counteract external loads and allow for deformation until the applied forces surpass the critical normal and tangential stress limits, resulting in bond failure and subsequent loss of cohesion between particles. The critical normal and tangential stress values are computed according to Equations 8, 9, respectively:


(8) $$\sigma_{max}<\frac{-F_{n}}{A}+\frac{2M_{t}}{J}R_{B}$$

(9) $$\tau_{max}<\frac{-F_{t}}{A}+\frac{M_{n}}{J}R_{B}$$

Where, $\sigma_{max}$ and $\tau_{max}$ are the critical normal stress and critical tangential stress.

In discrete element simulations, the multi-sphere method is a widely adopted technique for modeling irregularly shaped particles. To improve simulation accuracy and computational efficiency, a petiole model was established based on the natural growth morphology of radish petioles. Given the morphological characteristics of the white radish petiole, the petiole cross-section typically exhibits a U-shaped profile, with the concave side facing inward and the convex arc in direct contact with the clamping device. For the purpose of analyzing mechanical interactions between the petiole and the gripping mechanism, the cross-section was simplified as a circle with an equivalent radius, as shown in **Figure 10**. This simplification facilitates modeling under normal harvesting conditions, particularly for studying the biomechanical behavior of the petiole during clamping and pulling operations. However, in specific scenarios such as petiole clogging during harvesting, stress concentrations at the clamping interface may arise, and the circular cross-section simplification might not sufficiently capture the actual biomechanical response.

**Figure 11:**
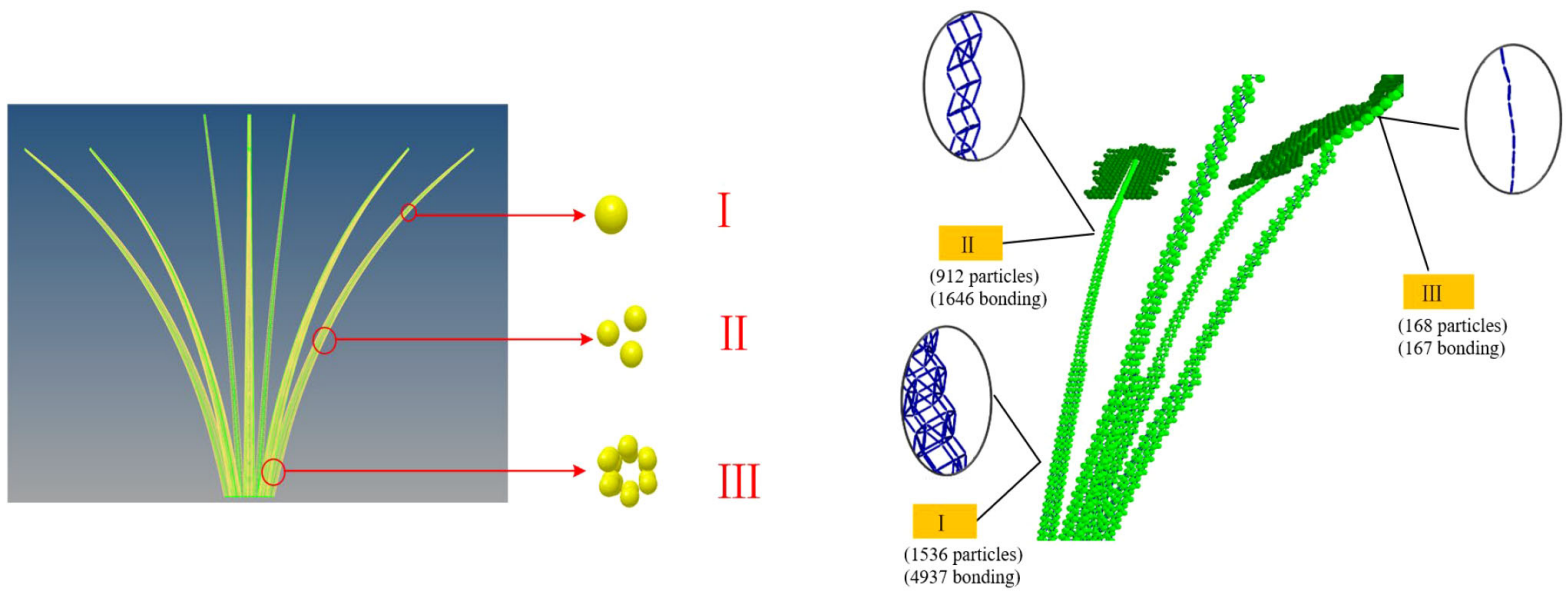
Discrete element model of radish petiole.

Additionally, during the construction of the BondingV2 model, traditional particle-filling methods could not be used to determine particle coordinates due to the requirement of avoiding inter-particle overlap. To construct a regularly arranged petiole model of the radish, the filled petiole model was imported into Hyperworks, where meshing was performed using Solidmaps and Automesh. A multi-scale modeling approach was adopted, based on the mean measurements from the tip, middle, and end portions of the petiole. The mesh center coordinates were extracted and exported to Excel. These mesh center coordinates represent the particle coordinates, with the inscribed sphere radius corresponding to the particle radius. The inscribed sphere radius within the mesh is equivalent to the particle radius. The exported mesh coordinates were subsequently input into the granular particle parameters. The discrete element model of the petiole of white radish is shown in **Figure 11**.

### 3.2 Calibration of discrete element model pmarameters

#### 3.2.1 Determination of the intrinsic parameters of the radish petiole

The intrinsic parameters of the radish petiole include density, Poisson’s ratio, and shear modulus. The density of the radish petiole was measured using a direct-reading solid density meter (MH-300G, range: 0.01–300 g, accuracy: 0.001 g/cm³, Shanghai Yixin Scientific Instruments Co., Ltd.). The tensile test was conducted using a Wane Mechanical Testing Machine (CMT6000, range: 5N–10000N, accuracy: ± 1%, SANS Shenzhen Anruibo Technology Co., Ltd.) to determine Poisson’s ratio and shear modulus. The compression speed was set to 3 mm/min, the displacement was set to 5 mm, and the changes in height and diameter of the material during the uniaxial compression test were measured. The Poisson’s ratio was calculated according to Equation 10, with the shear modulus considered equivalent to the torsional modulus. The resulting average density of the radish petiole was determined to be 862 kg/m³, while the Poisson’s ratio and shear modulus were found to be 0.27 and 1.34 × 10^6^ Pa, respectively.


(10) $$\mu =\frac{\left|\epsilon \right|}{\left|\epsilon^{\prime }\right|}=\frac{\left|l^{\prime }-l\right|}{\left|L^{\prime }-L\right|}$$

Where, μ is Poisson’s ratio, ϵ is the lateral deformation of the material (mm), $\epsilon^{\prime }$ is the longitudinal deformation of the material (mm), $l^{\prime }$ is the lateral length of the material before loading(mm), l is the lateral length of the material after loading(mm), $L^{\prime }$ is the longitudinal length of the material before loading(mm), L is the longitudinal length of the material after loading(mm).

#### 3.2.2 Determination of the intrinsic parameters of the radish petiole

**Figure 12:**
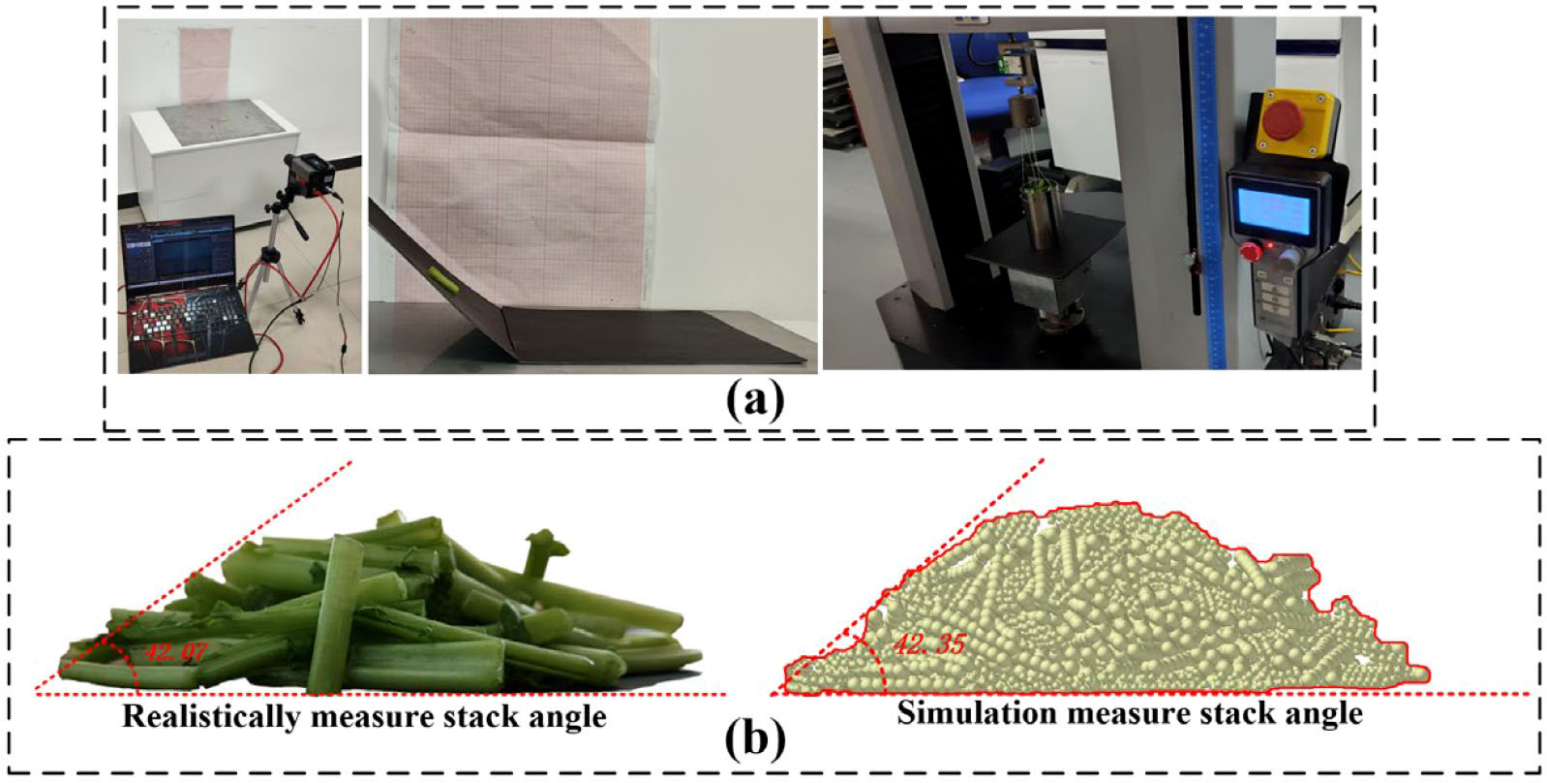
Calibration of contact parameters for the radish petiole. **(a)** Calibration experiment process for the radish petiole. **(b)** Validation experiment for the stack angle.

The collision restitution coefficient, static friction coefficient, and dynamic friction coefficient between the petiole-petiole and petiole-rubber clamping device were determined through free-fall and inclined-plane experiments. Additionally, the angle of repose of the radish petiole was determined using a cylindrical lifting test. Using the angle of repose as the evaluation criterion, the measured parameters were validated via a central composite design experiment, as shown in **Figure 12**. Ultimately, the contact parameters between the petiole-petiole and petiole-rubber clamping device were obtained, as detailed in **Table 2**.

#### 3.2.3 Determination of the bonding parameters for the discrete element model

Within the BondingV2 contact model, five key parameters influence the mechanical behavior of the radish petiole, including normal stiffness per unit area, shear stiffness per unit area, normal strength coefficient, shear strength coefficient, and the *Bonded Disk Scale* parameter. A three-point bending simulation was conducted using the radish petiole model, and the experimental data were processed using Design-Expert 13 software. Five discrete element bonding parameters were selected as experimental factors: normal stiffness per unit area (X_1_), shear stiffness per unit area (X_2_), normal strength coefficient (X_3_), shear strength coefficient (X_4_), and *Bonded Disk Scale* (X_5_). The three-point bending force (F_be_) was used as the response variable to analyze the influence of each factor. A steepest ascent experiment was then performed on the selected significant parameters to approach their optimal value ranges. Subsequently, the optimal contact parameters were determined based on a Plackett–Burman (PB) design. The factor levels used in the experimental design are summarized in **Table 3**.

**Table 3:** Experimental design factor levels table.

Parameter	Symbol	Parameter levels	
		Low	High
Normal stiffness per unit area/(N·m^-3^)	X_1_	1.0×10^9^	5.0×10^9^
Shear stiffness per unit area/(N·m^-3^)	X_2_	2.5×10^9^	7.5×10^9^
Normal strength/(Pa)	X_3_	1×10^11^	2×10^11^
Shear strength/(Pa)	X_4_	5×10^10^	1×10^11^
Bonded disk scale/(mm)	X_5_	1	1.5

**Figure 13:**
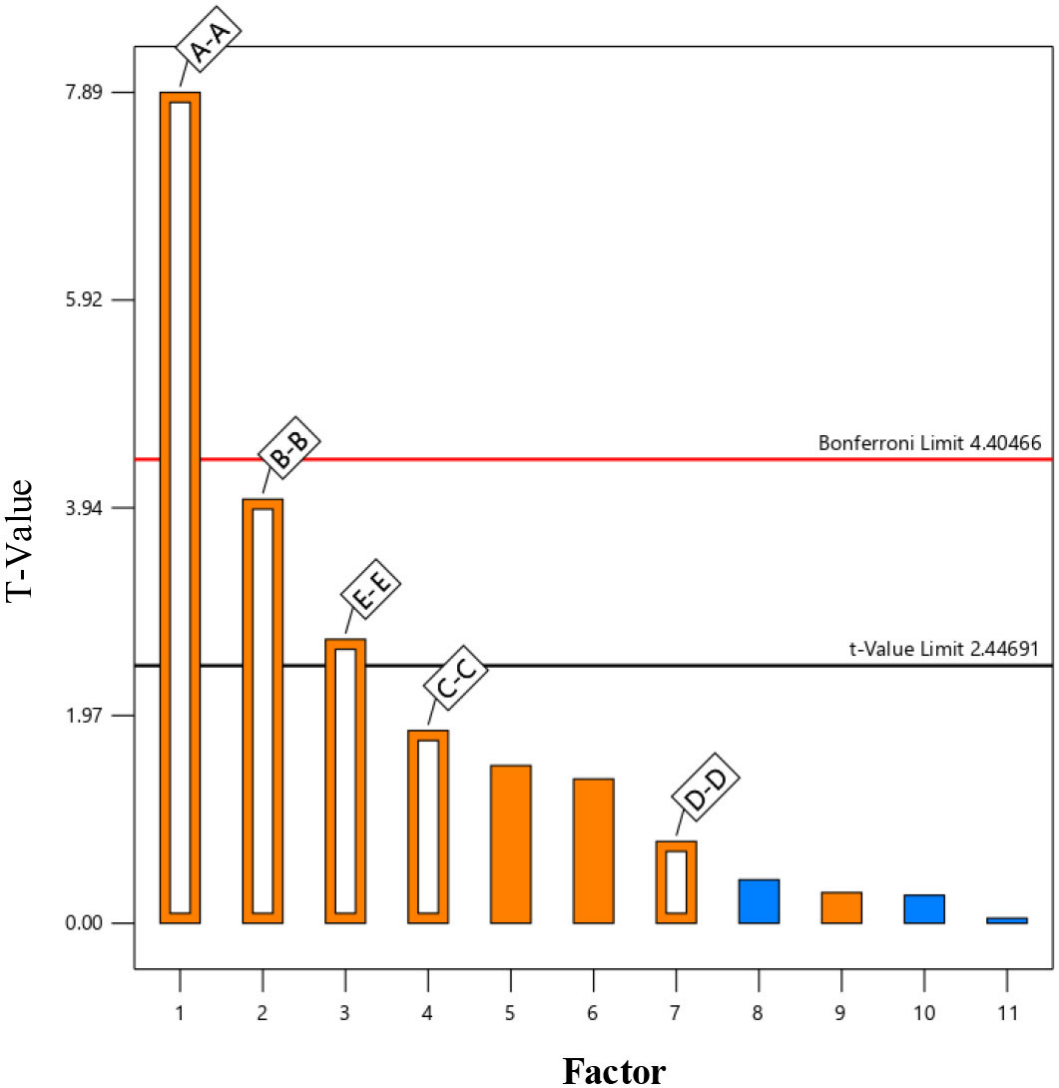
Pareto chart of standardized effects.

In this study, the Plackett-Burman module of Design-Expert software was employed to evaluate the effects of five factors on the three-point bending force (F_be_) using response values. Each factor was set at two levels (high and low), resulting in 12 experimental trials. The experimental design and results are shown in **Table 4**. A variance analysis was performed on the experimental data, and the Lenth method was applied to identify the significant effects in the Plackett-Burman experimental results. A Pareto chart of the standardized effects of the factors was generated, as depicted in **Figure 13**. The factors of normal stiffness per unit area (X_1_), shear stiffness per unit area (X_2_), and *Bonded Disk Scale* (X_5_) were found to have P-values less than 0.05, indicating their significant influence on the response. The significant factors affecting the angle of repose were determined to be normal stiffness per unit area (X_1_), shear stiffness per unit area (X_2_), and *Bonded Disk Scale* (X_5_).

**Table 4:** Plackett-Burman experimental design and results.

Group	*X_1_*	*X_2_*	*X_3_*	*X_4_*	*X_5_*	Three-point bending force F_be_/(N)
1	1	1	-1	1	1	11.38
2	-1	1	1	-1	1	7.82
3	1	-1	1	1	-1	8.53
4	-1	1	-1	1	1	6.69
5	-1	-1	1	-1	1	6.77
6	-1	-1	-1	1	-1	4.98
7	1	-1	-1	-1	1	7.91
8	1	1	-1	-1	-1	8.89
9	1	1	1	-1	-1	9.63
10	-1	1	1	1	-1	6.85
11	1	-1	1	1	1	9.22
12	-1	-1	-1	-1	-1	4.89

The P-value of the model is 0.0015, which is less than 0.05, indicating that the model is reliable and fits well within the regression region. The multiple correlation coefficient (R²) is 0.9371, suggesting a strong correlation. The adjusted R² value of 0.89 indicates that 89% of the variability in the experimental data can be explained by the regression model. Through multiple regression analysis, the following regression equation was obtained, as shown in Equation 11:


(11) $${\text{F}}_{\text{be}}=1.46{\text{X}}_{1}+0.747{\text{X}}_{2}+0.34{\text{X}}_{3}+0.145{\text{X}}_{4}+0.5{\text{X}}_{5}+7.8$$

Steepest ascent testing and response surface design were conducted for factors X_1_, X_2_ and X_5_, with X_3_ and X_4_ set to their middle values in subsequent experiments. In the regression equation, the coefficients of X_1_, X_2_ and X_5_ are all positive, indicating that these factors have a positive effect on the response angle. The steepest ascent test was performed with the relative error (R*_f_*) as the objective value.

The three-point bending force (F_b_) was measured experimentally to be 7.32 N. The relative error (R*_f_*) between the simulated three-point bending force (F_be_) and the actual measured force (F_b_) is given by, as shown in Equation 12:


(12) $$R_{f}=\frac{\left|F_{b}-F_{be}\right|}{F_{b}}\times 100\%$$

Where, R*_f_* is the relative error between the simulated and actual three-point bending forces (%); F_b_ is the actual three-point bending force measured through physical experiments (N); and F_be_ is the simulated three-point bending force (N).

As shown in **Table 5**, with the increase in factors X_1_, X_2_, and X_5_, the simulated three-point bending force gradually increases. The second group exhibits the smallest error (3.27%), and thus, the second group is chosen as the zero level, the first group as the low level, and the third group as the high level. A central composite design (CCD) experiment was conducted with the angle of repose as the response variable to determine the optimal parameter combination. The range of values for normal stiffness per unit area is 1×10^9^ to 3×10^9^, the range for shear stiffness per unit area is 2.5×10^9^ to 5×10^9^, and the range for Bonded Disk Scale is 1 to 1.25.

**Table 5:** Plackett-Burman experimental design and results.

Group	X_1_	X_2_	X_5_	Simulated bending force Fbe/(N)	Relative error R*_f_*/%
1	1×10^9^	2.5×10^9^	1	1	11.38
2	2×10^9^	3.75×10^9^	1.125	-1	7.82
3	3×10^9^	5×10^9^	1.25	1	8.53
4	4×10^9^	6.25×10^9^	1.375	1	6.69
5	5×10^9^	7.5×10^9^	1.5	-1	4.89

Based on the Plackett-Burman experiment and the steepest ascent test, factors X_1_, X_2_, and X_5_ were found to have a significant impact on the three-point bending force of the petiole. A central composite design (CCD) experiment was used to determine the optimal parameter combination. The factor codes for the experimental design are shown in **Table 6**, and the experimental scheme and results are presented in **Table 7**.

**Table 6:** Encoding table for central composite design experiment.

Code	Factors		
	X_1_	X_2_	X_5_
-1.414	3.18×10^8^	1.65×10^9^	0.91
-1	1×10^9^	2.5×10^9^	1
0	2×10^9^	3.75×10^9^	1.13
1	3×10^9^	5×10^9^	1.25
1.414	3.68×10^9^	5.85×10^9^	1.36

**Table 7:** Results of CCD experiment for three-point bending force of petiole.

Group	X_1_	X_2_	X_5_	Three-point bending force/(N)	Relative error R*_f_*/(%)
1	1×10^9^	2.5×10^9^	1	6.88	6.01%
2	3×10^9^	2.5×10^9^	1	7.00	4.37%
3	1×10^9^	5×10^9^	1	8.08	10.38%
4	3×10^9^	5×10^9^	1	8.12	10.93%
5	1×10^9^	2.5×10^9^	1.25	7.04	3.83%
6	3×10^9^	2.5×10^9^	1.25	7.67	4.78%
7	1×10^9^	5×10^9^	1.25	7.85	7.24%
8	3×10^9^	5×10^9^	1.25	8.49	15.98%
9	3.18×10^8^	3.75×10^9^	1.125	7.25	0.96%
10	3.68×10^9^	3.75×10^9^	1.125	7.87	7.51%
11	2×10^9^	1.65×10^9^	1.125	7.00	4.37%
12	2×10^9^	5.85×10^9^	1.125	8.34	13.93%
13	2×10^9^	3.75×10^9^	0.91	7.85	7.24%
14	2×10^9^	3.75×10^9^	1.34	7.86	7.38%
15	2×10^9^	3.75×10^9^	1.125	7.63	4.23%
16	2×10^9^	3.75×10^9^	1.125	7.40	1.09%
17	2×10^9^	3.75×10^9^	1.125	7.43	1.5%
18	2×10^9^	3.75×10^9^	1.125	7.38	0.82%
19	2×10^9^	3.75×10^9^	1.125	7.51	2.6%
20	2×10^9^	3.75×10^9^	1.125	7.45	1.78%

The experimental results were analyzed using variance analysis in Design-Expert software, as shown in **Table 8**. The P-value for the quadratic regression model of the three-point bending force is less than 0.01, while the P-value for the lack of fit term is 0.22. The model’s coefficient of determination (R²) is 0.97. The regression model is highly significant, the lack of fit term is not significant, and the high R² value indicates that the regression equation for the three-point bending force model fits well. The linear terms for X_1_, X_2_, and X_5_, as well as the interaction term between X_1_ and X_5_, and the quadratic term for X_5_, are all significant, while the remaining terms are not. The regression model for the three-point bending force and the factors is given by Equation 13.

**Table 8:** Analysis of variance for central composite design experiment.

Source	Sum of Squares	Degrees of Freedom	Mean Square	F-value	P-value	Significance
Model	3.8	9	0.4222	31.42	<0.01	**
*X_1_*	0.4477	1	0.4477	33.33	<0.01	**
*X_2_*	2.82	1	2.82	209.77	<0.01	**
*X_5_*	0.0713	1	0.0713	5.31	0.04	*
*X_1_X_2_*	0.0006	1	0.0006	0.0456	0.84	
*X_1_X_5_*	0.154	1	0.154	11.46	0.01	**
*X_2_X_5_*	0.0595	1	0.0595	4.43	0.06	
*X_1_^2^*	0.0051	1	0.0051	0.3765	0.55	
*X_2_^2^*	0.0479	1	0.0479	3.56	0.09	
*X_5_^2^*	0.2181	1	0.2181	16.24	<0.01	**
Lack of Fit	0.0922	5	0.0184	2.19	0.2051	
Pure Error	0.0421	5	0.0084			
Cor Total	3.93	19				

"*" denotes statistical significance at the 0.05 level; "**" indicates statistical significance at the 0.01 level.


(13) $$O=7.47\;+\;0.18X_{1}+0.45X_{2}+0.07X_{5}-0.01X_{1}X_{2}+0.14X_{1}X_{5}-0.09X_{2}X_{5}+0.02{X_{1}}^{2}+0.06{X_{2}}^{2}+0.12X_{5}2$$

**Figure 14:**
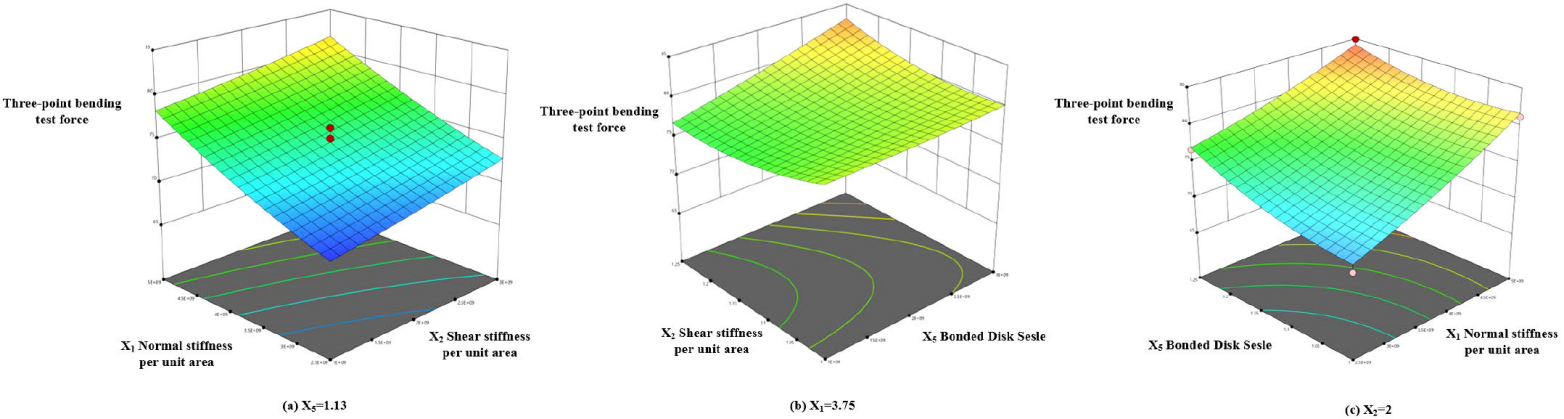
Response surface of the angle of repose experiment.

Based on the regression model in Equation 13, a response surface for the bonding parameters was constructed, as shown in **Figure 14**. The steepness of the response surface reflects the influence of interaction terms on the response value. The steeper the surface, the more significant the effect. The response surface plot shows that the three-point bending force increases gradually with the increase in factors X_1_, X_2_, and X_5_.

Using the optimization function in Design-Expert software, the regression model was optimized based on the measured three-point bending force (7.32 N), with the relative error set as the objective for optimization. The optimized values were then used to perform a three-point bending simulation. Based on the results of the central composite design and the regression equation, an optimal solution analysis was carried out for factors X_1_, X_2_, and X_5_. The objective function and constraint conditions are defined in Equation 14:


(14) $$\left\{ \begin{array}{c} \min R_{f}(X_{1},X_{2},X_{5})=0\\ s.t.\{\begin{array}{c} -1.414<X_{1}<1.414\\ -1.414<X_{2}<1.414\\ -1.414<X_{5}<1.414 \end{array} \end{array}\right.$$

Based on the constraint-solving tool in Design-Expert, as defined by Equation 14, the unit area normal stiffness (X_1_) coefficient for the radish petiole was determined to be 2×10^9^, the unit area shear stiffness (X_2_) coefficient was 3.12×10^9^, and the Bonded Disk Scale (X_5_) coefficient was 1.17. These parameter values were taken as the optimal values for the bonding parameter validation experiment, with other parameters set to the mean values from the Plackett-Burman (PB) experiment.

## 4 Feasibility of discrete element model validation

### 4.1 Analysis and verification of tensile failure test

**Figure 15:**
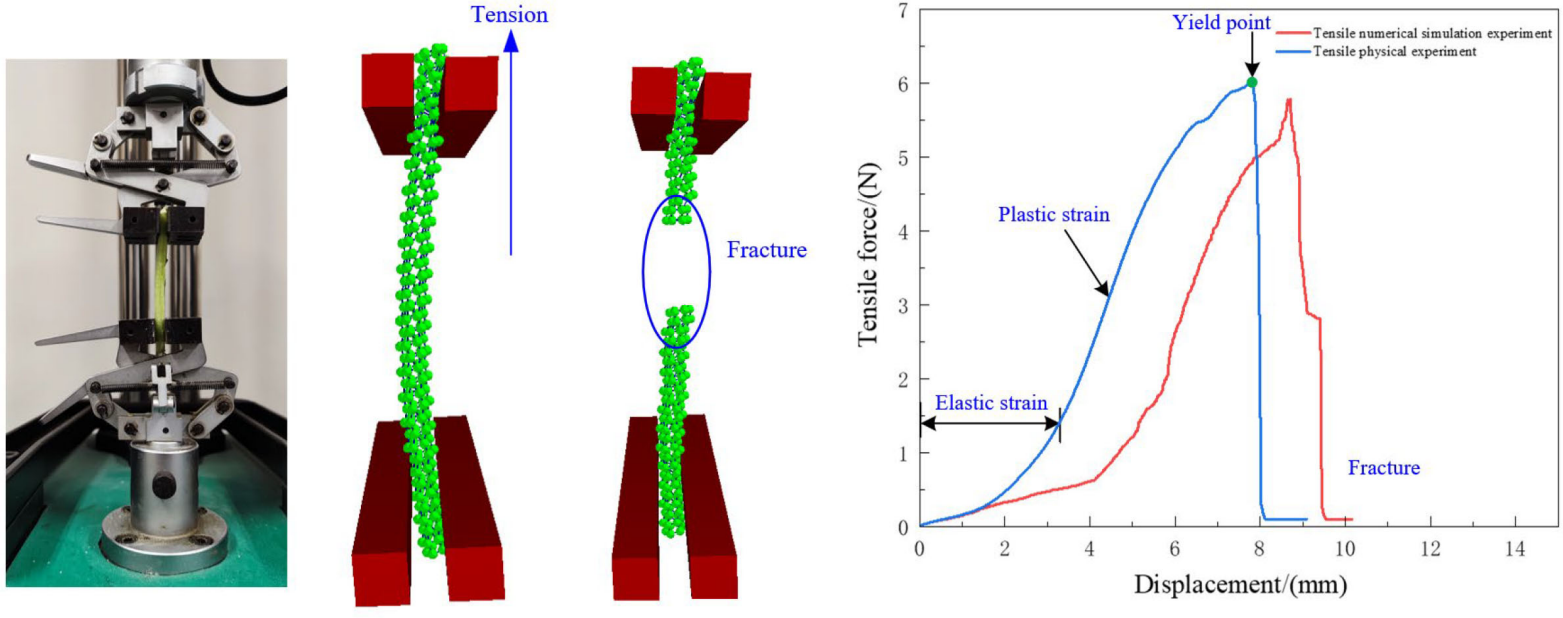
Petiole torsional failure test.

The lower fixture is fixed, while the upper fixture moves at a speed of 0.05 mm/s. As shown in **Figure 15**, the force-displacement relationship of the radish petiole indicates that the tensile fracture process can be divided into three stages: elastic deformation, tensile failure, and fracture. During the elastic deformation stage, the force and displacement are approximately linearly related and follow Hooke’s law, with force positively correlated to displacement. As the tensile force increases, the petiole’s tensile force and displacement remain linearly related until the yield point is reached. At this point, the vascular bundles and epidermal tissue of the petiole tear, and the tensile force rapidly decreases, marking the end of the test.

By comparing the curves of the simulation experiment with the actual experiment, it can be observed that the maximum tensile failure force from the simulation is slightly lower than that from the actual experiment. This is because the actual petiole surface is smoother, which requires a larger tensile force during the actual stretching process. Overall, the trends of the two curves are consistent. The maximum tensile force obtained from the simulation is 5.78 N, which differs by 4.46% from the target value of 6.05 N. This indicates that the discrete element model for the radish petiole effectively describes the relationship between tensile force and displacement.

The correlation coefficient is commonly used to evaluate the accuracy and reliability of a simulation model compared with experimental test data. To further verify the accuracy of the developed discrete element model in simulating tensile behavior, we analyzed the tensile force–displacement curves obtained from both simulation and physical experiments using Origin software. The specific method was as follows: 500 data points before the maximum tensile force were extracted from both the simulation and experimental datasets. Covariance and standard deviation were calculated, and the correlation coefficient R_k_ was computed based on the following formula:

The formula for calculating R_k_​ can be expressed as shown in Equation 15:


(15) $$R_{k}=\frac{\text{Cov}(X,Y)}{\sigma_{X}\sigma_{Y}}$$

In the equation, R_k_ represents the correlation coefficient between the simulation and experimental values, Cov(X,Y) denotes the covariance between the two datasets, $\sigma_{X}$ is the standard deviation of the simulation data, and, $\sigma_{Y}$ is the standard deviation of the experimental data.

The correlation coefficient R_k_ indicates the degree of similarity between the two curves: the closer R_k_ is to 1, the higher the similarity between the curves, suggesting that the simulated tensile force closely matches the actual experimental tensile force. A correlation coefficient close to 0 indicates little to no correlation, while a negative value suggests a negative correlation between the two curves. Based on this method, the calculated correlation coefficient was R_k_ = 0.97, demonstrating a high degree of consistency between the simulation and experimental curves. This result further validates the accuracy and reliability of the discrete element model developed in this study for describing the tensile behavior of radish petioles.

### 4.2 Analysis and verification of torsional failure test

**Figure 16:**
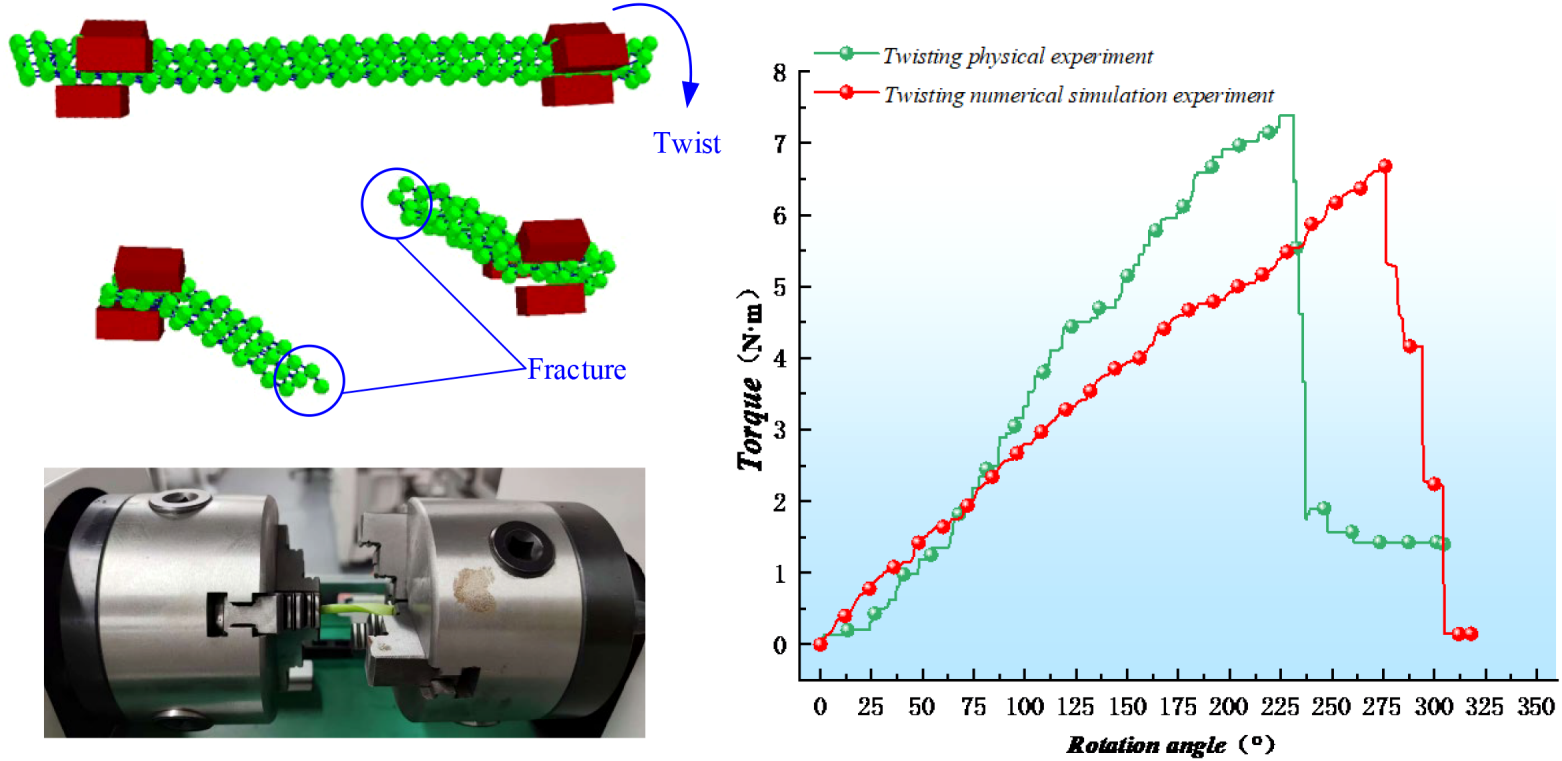
Petiole torsion failure test.

**Figure 16** presents the experimental and simulation results for the torsional failure of the radish petiole. Based on the calibrated parameters, the relationship between the petiole’s angle of rotation and torque was recorded in the simulation. Compared with the actual physical experiment, the torsional modulus of the simulated petiole is 1.26 MPa, while the torsional strength at the petiole’s end, as measured in the physical experiment, is 1.34 MPa, with a relative error of 5.97%. This indicates a good fit. The maximum torsional torque obtained from the simulation is 6.73 N·mm, at which point the bonding bond fractures. The maximum torsional force measured in the physical experiment is 7.38 N·mm, with a relative error of 8.8%, which is within an acceptable range.

### 4.3 Analysis and verification of three-point bending test

**Figure 17:**
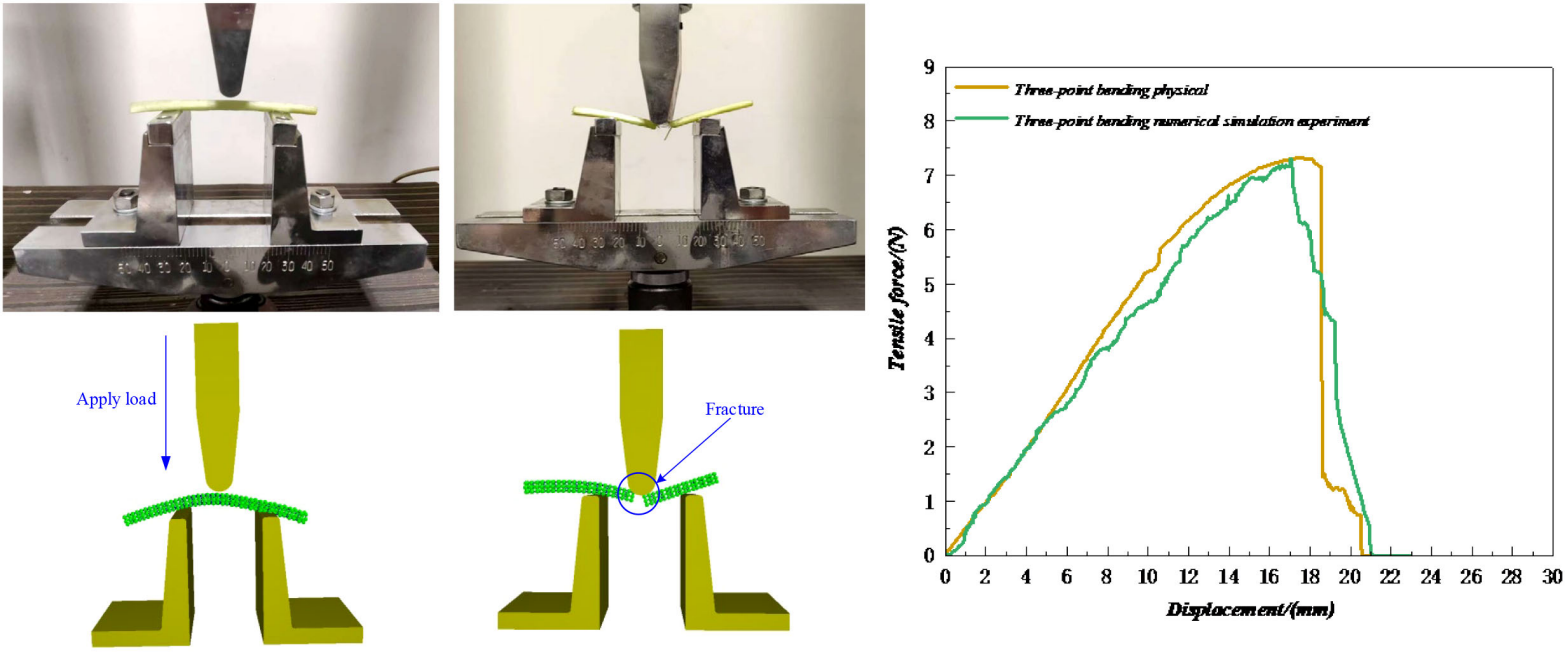
Three-point bending failure test.

**Figure 18:**
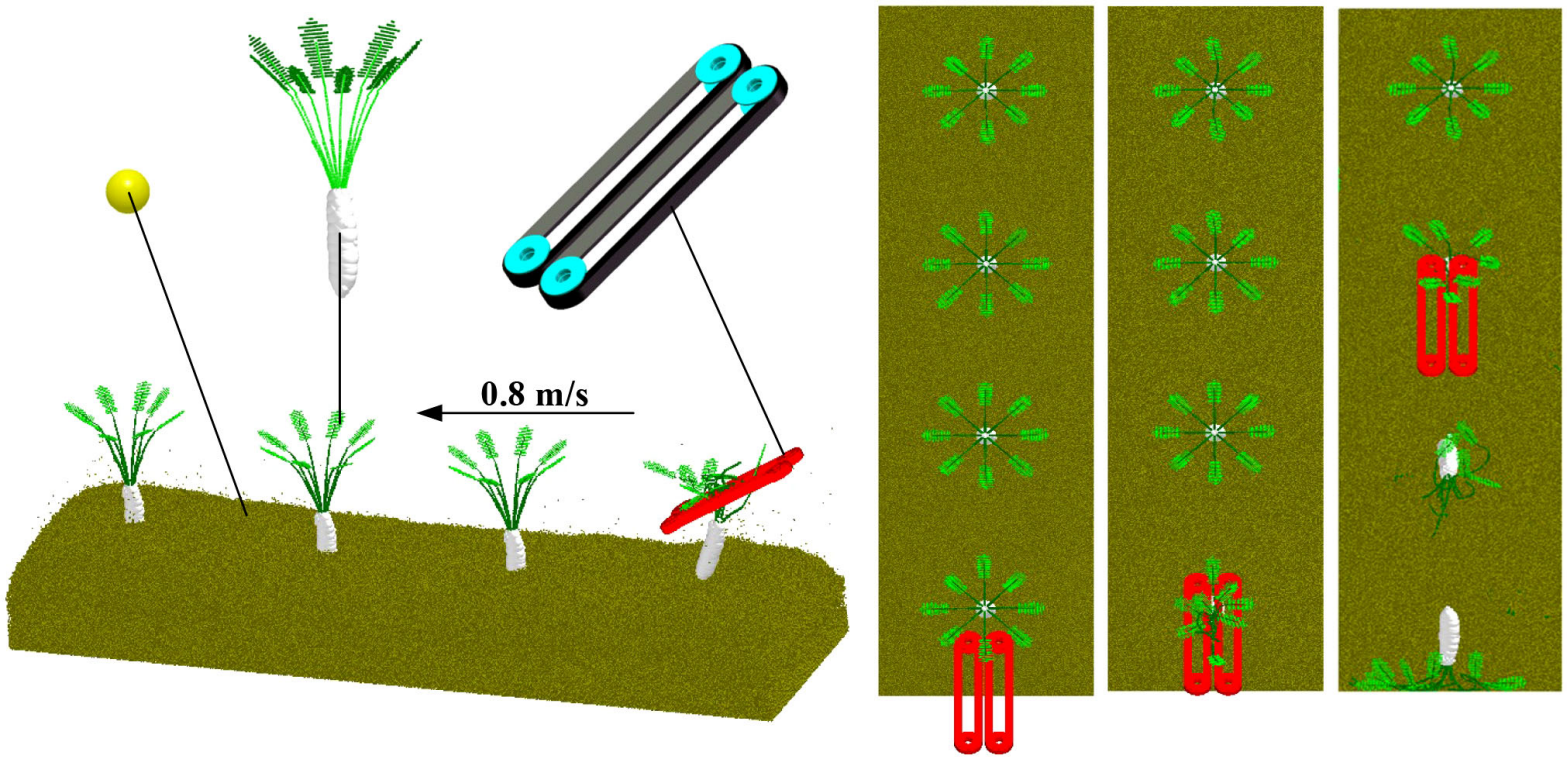
Simulation analysis of the petiole pulling process during radish harvesting.

In the three-point bending test, the petiole’s end was used as the material, and prior to the test, it was ensured that the petiole was intact with no tissue damage. As shown in **Figure 17**, the instruments were calibrated and zeroed before the test, and the test position was adjusted so that the pressure head moved downward at a constant speed of 5 mm/s until the petiole was bent and no rebound force was observed. After the petiole was bent, its tissue was damaged, and the entire process went through the stages of elastic deformation and plastic deformation. After the plastic deformation phase, the petiole tissue was essentially destroyed. As shown in **Figure 18**, this is the three-point bending deformation curve of the petiole. According to the data, the three-point bending failure force in the simulation experiment was 7.29 N, which differs by 0.41% from the actual measured value of 7.32 N, indicating a good simulation result.

### 4.4 Field test verification

Based on the established radish petiole model, a soil-radish root-petiole-clamping device simulation verification model was developed to replicate real field harvesting conditions. The breakage rate was used as an evaluation index to verify the calibration results of the petiole bonding parameters. Since there is no interaction between the petiole and the radish root, or between the soil and the clamping device during actual harvesting, the contact parameter calibration between the petiole and the root was omitted.

The discrete element method (DEM) was used to simulate the radish harvesting test. A model was built in EDEM software, generating 1,030,000 soil particles within a soil bin measuring 800 mm × 800 mm × 300 mm. A simplified rubber clamping device model was also established and imported into EDEM. The clamping device was set with a working tilt angle of 30°, a clamping wheel rotational radius of 109 mm, and a belt linear speed of 0.9 m/s. The forward speed of the clamping device was set along the negative Y-axis at 0.8 m/s, consistent with the actual field harvester’s speed. The radish harvesting simulation test is shown in **Figure 18**.

**Figure 19:**
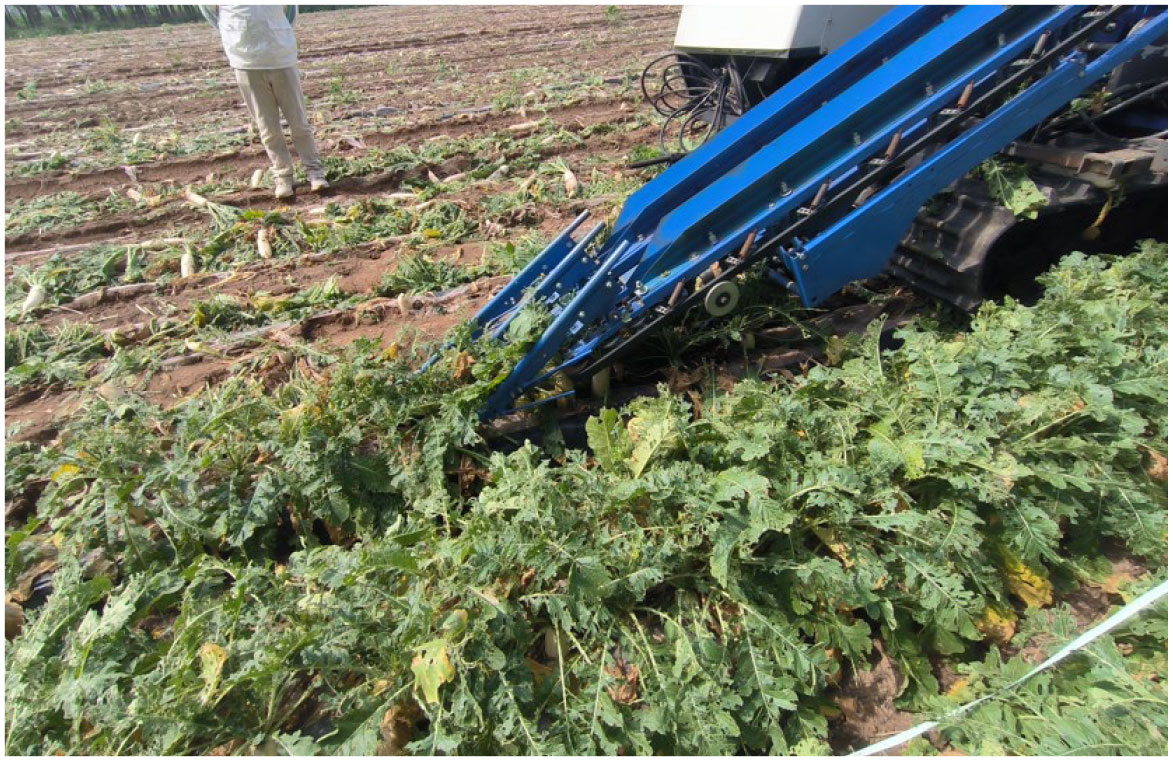
Field experiment on the petiole extraction process in radish harvesting.

**Figure 20:**
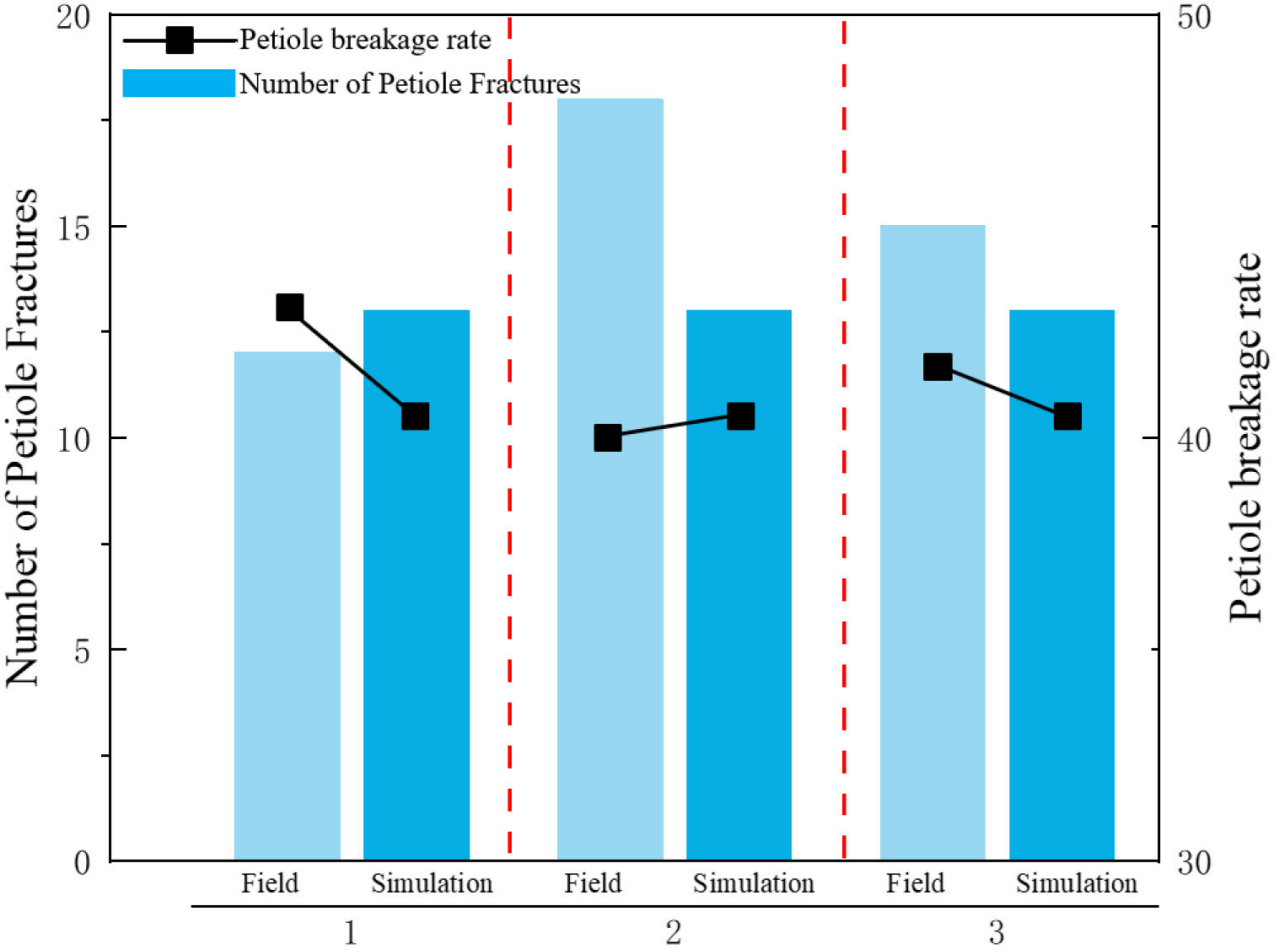
Results of field experiments.

**Figure 19** shows the actual field test. As shown in **Figure 20**, field test results indicate that when the equipment’s forward speed is 0.8 m/s, the working tilt angle is 30°, and the belt rotational linear speed is 0.9 m/s, the measured breakage rate is 41.5%, while the simulated breakage rate is 40.63%, resulting in a relative error of 2.1%. This demonstrates that the calibrated bonding parameters of the radish petiole can effectively characterize the radish discrete element model.

## 5 Conclusion

1. This study investigates the mechanical properties of radish petioles during the harvest period, and demonstrates that the torsional modulus is significantly influenced by petiole position, moisture content. The torsional modulus and maximum torque fracture value gradually decrease from the end to the tip, with the end showing the highest torsional modulus. This is mainly due to tissue structure differences, especially the greater proportion of vascular bundles at the petiole base. Therefore, during the harvesting process, prioritizing clamping at the base of the petiole can reduce the risk of damage. Furthermore, the torsional modulus exhibits a trend of first increasing and then decreasing with rising moisture content. Selecting the petiole base at an appropriate moisture level for gripping and extraction is thus essential for improving the efficiency and reliability of radish harvesting. This model is developed to investigate the force conditions and operational quality of a clamping-and-pulling-type radish harvester during the radish harvesting process. Therefore, it is specifically applicable to the mechanized harvesting of white radish. For other radish varieties(e.g., red radish, green radish), it is recommended to recalibrate the bonding parameters to account for the differences in biomechanical properties among crop types.
2. Based on the Hertz-Mindlin contact model and the BondingV2 bonding model, a discrete element model for the radish petiole was established. The optimal parameter combinations for the significant factors were determined through Plackett-Burman design and central composite experiments: the petiole’s unit area normal stiffness coefficient is 2×10^9^, unit area shear stiffness coefficient is 3.12×10^9^, normal strength is 1.5×10¹¹ Pa, shear strength is 7.5×10¹^0^ Pa, and the Bonded Disk Scale coefficient is 1.17.
3. Based on the above parameter combinations, axial tensile, torsional bending, three-point bending, and field tests were performed on the radish petiole model. The simulation results show errors of 4.46%, 8.8%, 0.41%, and 2.1% compared to the physical test results, with the simulation trend closely matching the physical test curves.

## Data Availability

The raw data supporting the conclusions of this article will be made available by the authors, without undue reservation.
